# Intercepting hepatocellular carcinoma in chronic hepatitis B: a structured critical review of Chinese herbal medicine and the immune–fibrotic evidence

**DOI:** 10.3389/fimmu.2026.1985689

**Published:** 2026-10-30

**Authors:** Jingjing Yang, Lihui Liu, Yingjie Liu, Wen Zhang, Honglin Chen, Tianze Gao, Baoping Luo, Xiaodong Li, Yana Zhou

**Affiliations:** 1 School of Traditional Chinese Medicine, Hubei University of Chinese Medicine, Wuhan, China; 2 Department of Oncology, Hubei Provincial Hospital of Traditional Chinese Medicine, Wuhan, China; 3 Hubei Shizhen Laboratory, Wuhan, China; 4 Department of Oncology, Affiliated Hospital of Hubei University of Chinese Medicine, Wuhan, China; 5 Institute of Liver Diseases, Hubei Key Laboratory of the Theory and Application Research of Liver and Kidney in Traditional Chinese Medicine, Wuhan, China

**Keywords:** cancer interception, Chinese herbal medicine, chronic hepatitis B, immune microenvironment, liver fibrosis

## Abstract

Patients with chronic hepatitis B (CHB) and advanced fibrosis or compensated cirrhosis remain at risk of incident hepatocellular carcinoma (HCC) despite antiviral therapy, creating a clinically relevant window for cancer interception. Chinese herbal medicine (CHM) combined with nucleos(t)ide analogs has been associated with fibrosis improvement and, in selected longitudinal studies, lower HCC incidence. This structured critical review asks whether CHM can intercept HCC by modifying actionable immune–fibrotic processes before tumor formation. We evaluated disease-side and CHM intervention evidence across macrophages/monocytes, T cells, natural killer cells, neutrophils, B cells, dendritic cells and myeloid-derived suppressor cells. Evidence maturity was assessed using pathological relevance, HCC linkage and temporal alignment, evidence level, sampling-compartment directness, functional validation and, for CHM evidence, translational potential. Specific CHM–immune-mechanism pairs were then mapped onto a four-tier interception framework. The T helper 17/regulatory T-cell axis (Th17/Treg) showed the clearest longitudinal human connection between fibrosis-associated immune imbalance and subsequent HCC. Macrophage and natural killer-cell programs were supported by cell-resolved fibrosis mechanisms, treatment-responsive changes and cross-stage HCC associations, whereas neutrophil evidence linked neutrophil extracellular traps with a less specific longitudinal neutrophil-to-lymphocyte-ratio signal. Evidence for B cells, dendritic cells and myeloid-derived suppressor cells was less continuous across disease stages. On the intervention side, epigallocatechin-3-gallate–HMGB1 (high-mobility group box 1)/NLRP3 (NLR family pyrin domain-containing 3) and matrine–NKG2D (natural killer group 2 member D) provided mechanistic evidence, while Fuzheng Huayu–macrophage NLRP3–hepatic stellate cell, Biejia-Ruangan–Th17/Treg and Fuzheng Huayu–lymphocyte–hepatic stellate cell combinations converged with formulation-level signals of reduced HCC incidence. However, no longitudinal cohort has linked defined CHM exposure, cell-resolved immune change, fibrosis trajectory and lower incident HCC risk within one time-ordered pathway. We therefore propose a minimum clinical–experimental design to test whether CHM-induced immune changes contribute to lower incident HCC risk.

## 1 Introduction

Chronic hepatitis B (CHB) remains a major cause of cirrhosis and hepatocellular carcinoma (HCC). Potent nucleos(t)ide analogs (NAs) durably suppress hepatitis B virus (HBV) replication, attenuate hepatic inflammation, and reduce the risks of hepatic decompensation and HCC (1, 2). In patients with established advanced fibrosis or compensated cirrhosis, however, antiviral therapy may be accompanied by incomplete structural recovery. Fibrosis can regress, stabilize, or progress, and a clinically meaningful residual risk of incident HCC persists even when liver stiffness improves during treatment (3–6). Current management therefore continues to rely on long-term antiviral therapy, repeated assessment of fibrosis and portal-hypertension-related complications, and sustained HCC surveillance (2, 7–9). A key unmet need is to determine whether adjunctive interventions can further remodel the fibrotic and immune processes that remain active during chronic HBV disease and thereby reduce incident HCC risk.

Cancer interception provides a clinically relevant framework for addressing this residual risk by targeting a biologically active pre-neoplastic period in high-risk individuals to prevent or delay clinically detectable cancer (10). In HBV-related liver disease, this pre-HCC state extends beyond fibrosis stage alone. Fibrogenesis emerges from reciprocal interactions among injured hepatocytes, hepatic stellate cells (HSCs) (11–17), extracellular matrix, and immune populations, with biological consequences determined by cell subset, functional state, anatomical compartment, and disease stage (18). Importantly, fibrosis is a shared wound-healing phenotype but not an immunologically uniform state. Single-cell studies have identified different intrahepatic monocyte/macrophage, T-cell, B-cell, and natural killer (NK) cell landscapes in HBV-related versus alcohol-related or non-viral cirrhosis, while metabolic dysfunction-associated steatotic liver disease (MASLD) and metabolic dysfunction-associated steatohepatitis (MASH) are characterized by distinct lipid-associated macrophage, immunoregulatory, and spatial fibrotic-niche programs (19–22). Human single-cell and tissue studies further demonstrate substantial discordance between circulating and intrahepatic immunity in CHB (23–27). Longitudinal liver sampling has shown treatment-responsive remodeling of intrahepatic CD8^+^ T-cell and macrophage programs during antiviral therapy (28–30). Together, these observations support a shift from viewing fibrosis only as a structural precursor of HCC toward defining HBV-specific, stage-associated immune states that may sustain or precede malignant progression.

This biological context provides the rationale for asking whether Chinese herbal medicine (CHM) can modify immune processes operating during HBV-related fibrosis and cirrhosis before HCC develops. Here, CHM encompasses multi-herb formulations, single herbs, herb pairs, herbal injections, standardized extracts, and chemically defined active constituents. A systematic review of 25 randomized trials involving 2,585 patients with HBV-related fibrosis or cirrhosis found that CHM combined with NAs was associated with changes in circulating T-cell and NK-cell indices, inflammatory mediators, and fibrosis-related outcomes (31). At the formulation level, randomized trials of Biejia-Ruangan (BJRG) and Ruangan Granule added to entecavir demonstrated histological regression of advanced fibrosis or early cirrhosis (32, 33). Extended follow-up of the Biejia-Ruangan trial and observational studies of Fuzheng Huayu (FZHY) further reported lower subsequent HCC incidence (34, 35), while a separate meta-analysis of CHM-containing regimens in HBV-related cirrhosis identified an overall signal toward reduced HCC occurrence (36). Collectively, these studies place CHM at the intersection of immune modulation, fibrosis remodeling, and HCC prevention and identify immune mediation of HCC-risk reduction as a key unresolved link.

The available literature can therefore be organized into two complementary evidence streams. The disease-side stream maps immune changes across HBV-related fibrosis, cirrhosis, and progression toward HCC to define candidate pre-HCC immune states. The intervention-side stream examines how CHM engages these immune populations and mechanisms and how such changes converge with antifibrotic and HCC outcomes. Clinical studies provide broad information on circulating lymphocyte subsets, NK-cell indices, and inflammatory mediators; mechanistic studies provide higher-resolution information on intrahepatic cell states, signaling pathways, and immune–stromal interactions; and longitudinal HCC studies provide outcome anchors for selected CHM interventions. Integration therefore requires two analytical levels: evidence maturity at the immune-cell-category level and progression of specific CHM–immune-target evidence chains toward cancer interception.

Accordingly, this structured critical review examines macrophage/monocyte, T-cell, NK-cell, neutrophil, B-cell, dendritic-cell, and myeloid-derived suppressor-cell programs across HBV-related fibrosis and cirrhosis and evaluates their engagement by CHM. Evidence maturity is characterized using paired disease-side and intervention-side profiles. Disease-side evidence is assessed across five dimensions: pathological relevance, HCC linkage and temporal alignment, evidence level, sampling-compartment directness, and functional validation. The intervention-side profile retains these domains, with pathological relevance serving as a shared biological anchor, and additionally evaluates translational potential. In parallel, specific CHM–immune-target combinations are mapped onto a four-tier evidence chain spanning disease-defined target identification, CHM-mediated target engagement, convergence with HCC-reduction evidence, and mechanistic closure of immune-mediated cancer interception. By integrating disease immunology, CHM immunopharmacology, antifibrotic outcomes, and incident HCC evidence, this review aims to identify biologically credible candidate targets, distinguish intervention-responsive mechanisms, prioritize pathways closest to cancer-interception translation, and define the evidence required to establish that CHM-induced immune remodeling contributes to lower incident HCC risk. The overall analytical framework is summarized in **Figure 1**.

**Figure 1:**
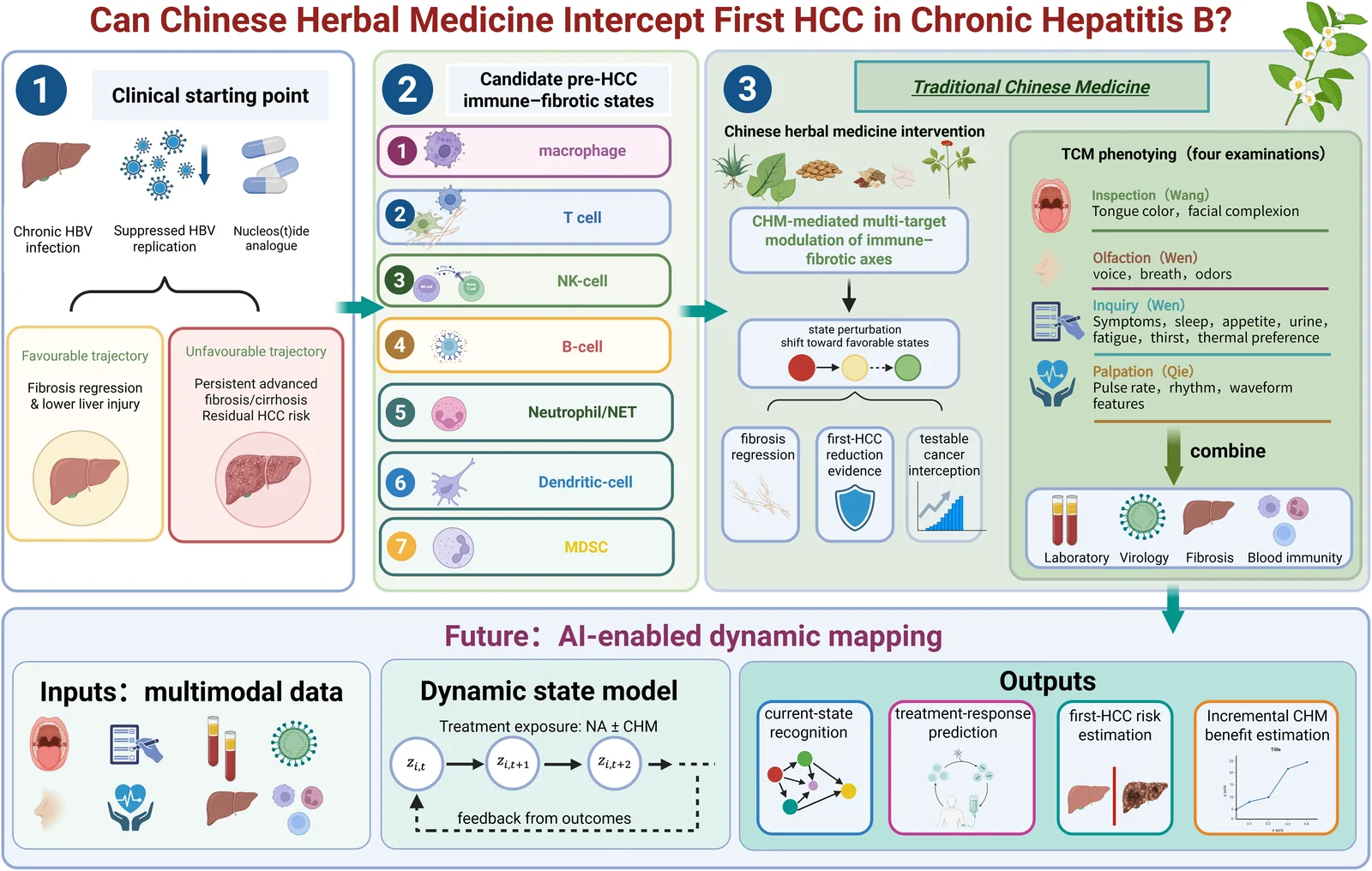
Conceptual framework for immune-mediated HCC interception in chronic hepatitis (B) The figure presents the conceptual rationale and overall research vision proposed in this review. The three upper panels outline the clinical setting of residual HCC risk during antiviral therapy, candidate pre-HCC immune–fibrotic states across seven immune-cell populations, and the potential role of CHM interventions. Future studies could incorporate exploratory TCM phenotyping and multimodal measurements to investigate these relationships. The lower panel depicts a possible future AI-enabled approach to integrating longitudinal data for state recognition, treatment-response prediction, incident-HCC risk estimation and assessment of incremental CHM benefit. These proposed applications require validation. In the dynamic model, z(i, t) denotes the proposed patient-level state of individual *i* at time *t*. Arrows indicate conceptual connections, proposed temporal transitions or analytical flow, rather than established causal effects; the feedback loop represents potential use of subsequent outcomes to refine the model. Incident HCC refers to newly diagnosed HCC after study entry in patients without baseline HCC, not recurrence. (AI, artificial intelligence; CHM, Chinese herbal medicine; HBV, hepatitis B virus; HCC, hepatocellular carcinoma; MDSC, myeloid-derived suppressor cell; NA, nucleos(t)ide analog; NET, neutrophil extracellular trap; NK, natural killer; TCM, traditional Chinese medicine). Created in BioRender. yang, J. (2026) https://BioRender.com/f9n8sit.

## 2 Evidence assessment framework

### 2.1 Review design and scope

This structured critical review critically appraises evidence that CHM modulates immune-cell responses in HBV-related liver fibrosis and compensated cirrhosis. The central questions are whether CHM can regulate specific immune-cell populations and functions involved in fibrotic or cirrhotic progression before HCC develops; whether these immune alterations are supported by intervention-responsive, temporally ordered and functional evidence; and whether they are further associated with a reduced risk of incident HCC.

### 2.2 Search strategy

We searched PubMed, Web of Science Core Collection, the China National Knowledge Infrastructure and Wanfang Data for English- and Chinese-language publications, focusing on work published between January 2010 and August 2026. Search terms covered HBV (“hepatitis B,” “HBV”), liver disease (“liver fibrosis,” “cirrhosis,” “hepatocellular carcinoma”), immune cells (macrophages/monocytes, T cells, natural killer cells, neutrophils, B cells, dendritic cells and myeloid-derived suppressor cells), and CHM (“Chinese herbal medicine,” “traditional Chinese medicine,” “Chinese patent medicine,” “herb-derived compound,” and specific formulation or constituent names). Terms were combined according to each search stream. Detailed strategies and date limits are provided in **Supplementary Table S1**. Evidence was synthesized narratively and critically appraised.

### 2.3 Inclusion and exclusion criteria

Inclusion criteria: Original studies examining immune mechanisms in HBV-related fibrosis/cirrhosis, including their associations with HCC; CHM-associated immune–fibrotic changes, or associations between CHM use and the risk of incident HCC. Eligible interventions included multi-herb formulations, single herbs, herb pairs, herbal injections, standardized extracts and chemically defined active constituents. Exclusion criteria: Duplicate records; studies unrelated to these domains; reviews, books, letters, conference papers, guidelines and unavailable full text; non-HBV model studies unrelated to the review scope; studies reporting only HCC recurrence or treatment of established HCC; and studies with insufficient reporting of study design, intervention regimens, or outcome assessment to permit meaningful appraisal were excluded from the core evidence synthesis. Two researchers, Wen Zhang (WZ) and Tianze Gao (TZG), independently screened titles and abstracts from the same deduplicated set of records and assessed the full texts of potentially eligible studies against the inclusion and exclusion criteria. Disagreements were adjudicated by a third researcher, Jingjing Yang (JJY).

### 2.4 Evidence rating and tier assignment

Evidence maturity was evaluated at the immune-cell-category level. Disease-side evidence was assessed across five prespecified dimensions, with translational potential added as a sixth dimension for CHM intervention evidence. First, pathological relevance assesses whether alterations in immune-cell abundance, phenotype or function occur directly in HBV-related fibrosis or cirrhosis and are associated with hepatic inflammation, HSC activation, extracellular matrix (ECM) remodeling or fibrosis progression. Second, HCC linkage and temporal alignment distinguishes mechanisms confined to fibrosis or cirrhosis from those supported by cross-sectional HCC associations, longitudinal pre-HCC observations, or experimental studies in which treatment is initiated before tumorigenesis and subsequent HCC outcomes are assessed. Third, evidence level considers whether evidence is derived from *in vitro* experiments, HBV-relevant animal models or human studies, thereby reflecting its proximity to the clinical setting of HBV-related liver disease. Fourth, sampling-compartment directness evaluates whether immune changes are measured in peripheral blood, isolated intrahepatic immune cells or liver tissue, and distinguishes systemic immunopharmacological effects from local intrahepatic mechanisms. Fifth, functional validation examines the use of cell-specific phenotyping or functional assays, as well as cell depletion, adoptive transfer, co-culture, pathway blockade, genetic manipulation or rescue experiments. Sixth, translational potential determines whether outcomes are restricted to molecular, cellular or circulating immune markers or extend to fibrosis biomarkers, liver stiffness, histological regression, cirrhosis-related clinical outcomes and HCC incidence. Scores of 0–3 denote no direct evidence and low, moderate, and high levels of evidentiary support, respectively (**Table 1**). Together, these dimensions define the evidence-maturity profile of each immune-cell population. Evidence was interpreted according to study design and measured endpoints, distinguishing disease-stage associations, treatment-associated immune changes and experimental mechanisms from evidence of immune-mediated HCC prevention.

**Table 1:** Six-dimensional evidence-maturity rubric for HBV-related immune-cell evidence.

Dimension	0 — None	1 — Low	2 — Moderate	3 — High
D1: Pathological relevance	No relevant evidence linking the immune mechanism to HBV-related fibrosis or cirrhosis.	Indirect, primarily antiviral, non-HBV, or systemic evidence; relevance to HBV fibrogenesis is uncertain.	Human HBV association or compatible functional evidence links the mechanism to fibrosis, but tissue anchoring, stage specificity, or consistency is limited.	Direct human HBV liver evidence links the defined immune mechanism to inflammation, HSC activation, ECM remodeling, fibrosis progression or cirrhosis.
D2: HCC linkage and temporal alignment	No HCC-related evidence.	Contextual HCC linkage only, such as a cross-sectional association of the same or closely related immune-cell state with established HCC.	Disease side: The defined immune mechanism measured before HCC is associated with subsequent incident HCC, without independent human replication. CHM side: The defined CHM intervention has longitudinal human or pre-HCC experimental evidence of lower subsequent HCC incidence.	Disease side: Human longitudinal evidence links the defined pre-HCC immune mechanism to subsequent incident HCC, with independent replication. CHM side: Human longitudinal evidence links CHM-induced immune changes to lower subsequent incident HCC risk, with independent replication.
D3: Evidence level	No relevant study evidence.	Evidence is limited to indirect proxies, non-HBV transfer evidence or mechanistic inference without direct testing of the immune mechanism.	Related animal/*in vitro* evidence, or a limited human study without complementary mechanistic validation.	Human studies directly evaluate the immune mechanism, with complementary ex vivo, animal or mechanistic validation.
D4: Sampling-compartment directness	No relevant immune measurements.	Peripheral/systemic measurements only, or ex vivo evidence without liver anchoring.	Direct human liver or HBV-animal liver measurement using a target-resolved assay, or paired systemic–tissue evidence that anchors the mechanism to the liver.	Repeated, longitudinally paired or spatially resolved human liver immune measurements directly characterize the assessed mechanism.
D5: Functional validation	No functional evidence.	Cell proportions, receptor/marker expression, cytokines or pathway abundance only, without causal validation.	Cell-specific functional assay, co-culture, target-cell assay or pharmacological inhibition demonstrates a functional consequence but not definitive mechanistic causality.	Depletion, adoptive transfer, genetic manipulation, pathway blockade or rescue demonstrates mechanistic dependency of the immune effect and relevant disease phenotype.
D6: Translational potential	No translational evidence.	Immune markers, symptoms, liver-injury indices or preclinical disease endpoints only.	Human fibrosis biomarkers, liver stiffness, histological changes or cirrhosis-related clinical outcomes are assessed alongside the relevant immune readouts.	Longitudinal human evidence shows that intervention-associated changes in the assessed immune mechanism precede and are associated with incident HCC or another major clinical outcome.

Scores range from 0 (none) to 3 (high): five dimensions apply to both evidence streams; the sixth applies only to CHM interventions. Across the applicable dimensions, disease-side ratings concern immune mechanisms in HBV-related liver fibrosis/cirrhosis and HCC development, whereas CHM-side ratings concern immune mechanisms associated with CHM interventions and related fibrosis and HCC outcomes. CHM-side ratings do not inherit disease-side scores. Dimension scores are not summed or equated with interception tiers. (CHM, Chinese herbal medicine; HBV, hepatitis B virus; HCC, hepatocellular carcinoma).

WZ and TZG independently assigned the dimensional scores using the prespecified anchors in **Table 1** and recorded the supporting references for each rating. Disagreements were initially resolved through discussion; unresolved cases were adjudicated by JJY. When evidence fell between two adjacent categories, the lower score was retained unless all criteria for the higher category were satisfied.

HBV-specific human studies and HBV-relevant models anchored disease attribution. Non-HBV models in disease-mechanism and CHM studies provided functional evidence within the tested system; their application to HBV-related fibrosis or HCC requires validation in HBV-relevant systems (11, 37, 38). Blood and liver measurements were interpreted separately, distinguishing cell abundance, phenotype, transcriptional state and function.

The specific intervention–immune mechanism pair is used as the fundamental unit of assessment. A four-tier threshold framework is then applied to classify the cancer-interception relevance of CHM-associated immune evidence (**Table 2**). Each pair was assigned to the highest tier for which all minimum criteria were satisfied; tier classification was not inferred by summing the dimensional scores. Application of the tier criteria is illustrated using the FZHY–macrophage NLRP3/HSC axis (NLRP3, NLR family pyrin domain-containing 3; **Table 3**).

**Table 2:** Four-tier framework for CHM-associated immune interception of incident HCC.

Tier	Recommended term	Minimum evidentiary gate	Core question	Interpretive boundary
Tier 1	Disease-anchored candidate immune target	A defined immune-cell state, subset, or mechanism is associated with HBV-related fibrosis or cirrhosis; repeated or longitudinal evidence supports trajectory designation.	Is this a biologically credible pre-HCC immune target?	Establishes a disease-side target and its interception priority.
Tier 2	CHM-responsive immune mechanism	A defined CHM intervention shifts the immune target in a favorable direction together with improvement in fibrosis, HSC activation, ECM remodeling, or histology.	Can CHM engage the target and improve the fibrotic phenotype?	Establishes immune target engagement by CHM.
Tier 3	Convergent-evidence cancer-interception candidate	Tier 2 evidence plus independent longitudinal human or pre-HCC experimental evidence that the same CHM intervention reduces subsequent HCC.	Does the same CHM intervention also have evidence of lower subsequent HCC incidence?	Indicates convergence of immune–fibrotic and HCC outcome evidence at the intervention level, without establishing immune mediation.
Tier 4	Validated immune-mediated cancer interception	Human longitudinal evidence establishes CHM exposure → specific immune change → pre-HCC/fibrotic remodeling → lower incident HCC risk, supported by mediation or mechanistic evidence and independent validation.	Does the immune change mediate the reduction in incident HCC risk?	Establishes mechanistic closure of immune-mediated cancer interception.

Pairs advance from a disease-anchored target (Tier 1) to CHM target engagement (Tier 2), outcome convergence (Tier 3) and human mechanistic closure (Tier 4). Tier denotes evidence-chain maturity and cannot be inferred by summing dimensional scores.

**Table 3:** Tiering example: macrophage–FZHY NLRP3/HSC axis.

Tier	Evidence supporting	Gate met
Tier 1	NLRP3-positive cells accumulated in association with collagen deposits in human HBV-related fibrotic liver tissue (39).	YES
Tier 2	FZHY reduced hepatic NLRP3 expression in patients with HBV-related fibrosis receiving entecavir. Complementary DDC-model and macrophage–HSC experiments supported suppression of macrophage NLRP3 activity, HSC activation and fibrosis (39).	YES
Tier 3	Retrospective cohorts reported lower subsequent HCC incidence with FZHY added to antiviral therapy in HBV-related cirrhosis (35, 40), complementing the Tier 2 evidence.	YES
Tier 4	The cited studies do not establish that FZHY-induced macrophage NLRP3 changes mediate lower subsequent HCC risk in humans, with independent validation.	NO

Final assignment: Tier 3. Tier assignment reflects the highest level for which all criteria in **Table 2** were met. The evidence converges across studies at the FZHY intervention level but does not establish that macrophage NLRP3 modulation mediates lower HCC incidence. (FZHY, Fuzheng Huayu; NLRP3, NLR family pyrin domain-containing 3; HSC, hepatic stellate cell; HBV, hepatitis B virus; CHM, Chinese herbal medicine; HCC, hepatocellular carcinoma; DDC, 3,5-diethoxycarbonyl-1,4-dihydrocollidine).

## 3 Immune microenvironment in HBV-related fibrosis and cirrhosis

HBV-related liver fibrosis develops within an immune environment shaped by persistent viral antigen exposure, hepatocyte injury and stromal activation, accompanied by dynamic remodeling of circulating and intrahepatic immune-cell populations. These changes are specific to cell type, anatomical compartment and disease stage, and the same immune population may exert antiviral, hepatotoxic, profibrotic or antifibrotic effects under different conditions. Thus, their biological significance depends not only on abundance but also on cellular subset, functional state and tissue localization. This review examines immune remodeling across the continuum of HBV-related fibrosis, cirrhosis and HCC, focusing on stage-associated trajectories and their relationships with fibrosis progression, treatment responsiveness and HCC development (**Figure 2**). **Table 4** summarizes representative studies of seven immune-cell categories, integrating disease stage, tissue compartment, functional evidence, study design and HCC linkage.

**Figure 2:**
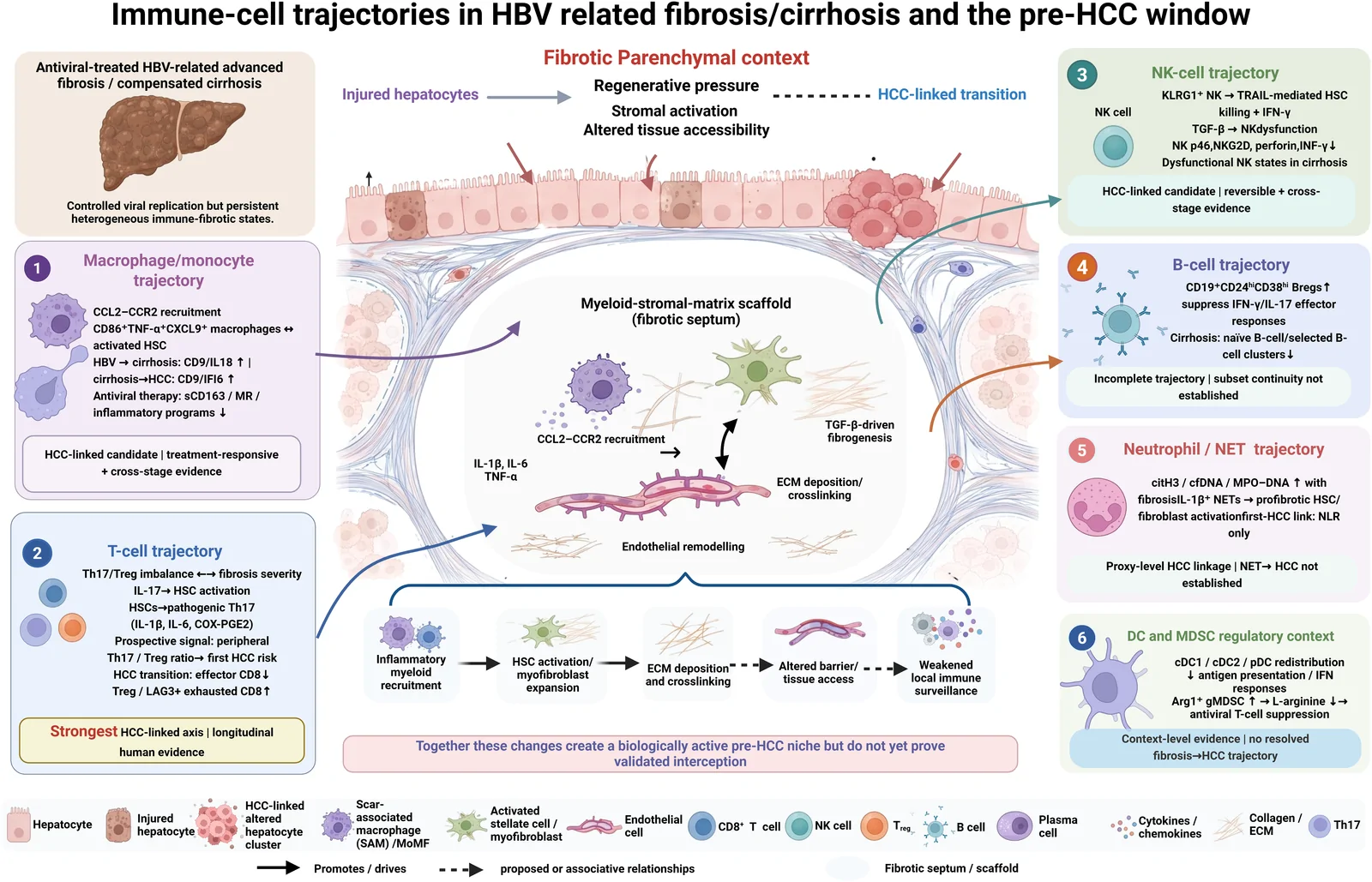
Immune-cell trajectories in HBV fibrosis/cirrhosis and the pre-HCC window. The central scaffold integrates myeloid recruitment, HSC activation, endothelial remodeling and ECM deposition, surrounded by panels summarizing disease-side evidence for seven immune-cell populations; DCs and MDSCs share one panel. “Trajectories” describes the organization of immune changes across disease stages, not uniformly validated longitudinal courses. The Th17/Treg axis has longitudinal human HCC-associated evidence, whereas macrophage and NK-cell HCC links are mainly cross-sectional. Neutrophil-associated HCC evidence relies on NLR as a composite proxy, and continuity across disease stages remains incomplete for B cells, DCs and MDSCs. Solid arrows indicate reported functional relationships, dashed links indicate proposed or associative relationships, and colored connectors link the scaffold to the corresponding immune-cell panels. Upward and downward arrows denote reported increases and decreases, respectively. (ARG1, arginase 1; Breg, regulatory B cell; CCL, C–C motif chemokine ligand; CCR, C–C chemokine receptor; CD, cluster of differentiation; cDC, conventional dendritic cell; cfDNA, cell-free DNA; citH3, citrullinated histone H3; COX, cyclooxygenase; CXCL, C–X–C motif chemokine ligand; DC, dendritic cell; ECM, extracellular matrix; HBV, hepatitis B virus; HCC, hepatocellular carcinoma; HSC, hepatic stellate cell; IFN, interferon; IL, interleukin; KLRG1, killer cell lectin-like receptor G1; LAG3, lymphocyte-activation gene 3; MDSC, myeloid-derived suppressor cell; MoMF, monocyte-derived macrophage; MPO–DNA, myeloperoxidase–DNA complex; MR, mannose receptor; NET, neutrophil extracellular trap; NK, natural killer; NKG2D, natural killer group 2 member D; NKp46, natural cytotoxicity receptor NKp46; NLR, neutrophil-to-lymphocyte ratio; pDC, plasmacytoid dendritic cell; PGE_2_, prostaglandin E_2_; SAM, scar-associated macrophage; sCD163, soluble CD163; TGF-β, transforming growth factor beta; Th17, T helper 17 cell; TLR, Toll-like receptor; TNF, tumor necrosis factor; TRAIL, TNF-related apoptosis-inducing ligand; Treg, regulatory T cell). Created in BioRender. yang, J. (2026) https://BioRender.com/f9n8sit.

**Table 4:** Representative evidence for immune-cell-associated mechanisms in HBV-related fibrosis and cirrhosis, stratified by HCC linkage.

Immune-cell population	Disease context/comparison/model	Main mechanism/pathway	Sampling compartment	Evidence system	Functional validation	Temporal design	HCC linkage	Ref.
Macrophages/monocytes	HBV-related fibrosis (METAVIR F0–F4)	Activated HSC-derived CCL2 recruits CCR2^+^ macrophages and promotes CD163^+^/CD206^+^ differentiation	Human liver	Human: histology + *in vitro*	macrophage–HSC co-culture; recombinant CCL2; CCR2 antagonism	Cross-sectional	None	(41)
Macrophages/monocytes	HBV-related fibrosis vs healthy controls	CD86^+^TNF-α^+^CXCL9^+^ M1-like macrophages colocalize with activated HSCs and collagen-rich niches; HBV-conditioned macrophages activate HSCs	Human liver + serum	Human: spatial profiling + *in vitro*	Spatial colocalization and macrophage–HSC co-culture	Cross-sectional	None	(42)
Macrophages/monocytes	HBV-related fibrosis vs healthy controls	Monocytes expand; ITGA2B^+^ monocyte–platelet aggregates show inflammatory, chemokine and endothelial-adhesion programs	Peripheral blood	Human: scRNA-seq + *in vitro*	Flow-cytometric validation; endothelial adhesion; LX-2 response assays	Cross-sectional	None	(43)
Macrophages/monocytes	HBV-related cirrhosis vs Healthy controls	Macrophage-CD9/IL18 and macrophage-C1QA states accumulate in cirrhotic liver	Human liver	Human: scRNA-seq	Subset annotation, pseudotime and inferred cell communication	Cross-sectional	None	(25)
Macrophages/monocytes	HBV vs HBV-related cirrhosis vs HBV-HCC vs Healthy controls	Macrophage-CD9/IL18 expands from infection to cirrhosis; macrophage-CD9/IFI6 is enriched from cirrhosis to HCC	Human liver	Human: scRNA-seq	Subset annotation and inferred cell communication	Cross-sectional	Cross-sectional HCC association	(18)
Macrophages/monocytes	HBV-related fibrosis (Ishak F0–F6)	Soluble CD163 and soluble mannose receptor track histological activity/fibrosis; hepatic CD163^+^ macrophages decline with therapy	Human liver + serum	Human: serum biomarkers + histology	Biomarker–histology correlation and hepatic CD163 immunohistochemistry; no cell-specific perturbation	Cross-sectional + 48-week NA-treatment follow-up	None	(44)
Macrophages/monocytes	HBV-related cirrhosis vs non-viral/alcohol-related cirrhosis vs Healthy controls	Macrophage-PLCG2 proportions are higher and macrophage-CD5L proportions lower in HBV-related cirrhosis compared with non-viral cirrhosis	Human liver	Human: scRNA-seq + spatial analysis; public single-cell; spatial datasets	Immunofluorescence hemic and spatial validation; no causal perturbation	Cross-sectional	None	(22)
Macrophages/monocytes	Human: HBV-related fibrosis/cirrhosis; Rat: CCl_4_/2-AAF-induced fibrosis	Macrophage Wnt5a–FZD2 signaling alters hepatic progenitor-cell fate and fibrogenesis	Human liver + rat liver	Human: histology; Rat: *in vivo* + *in vitro*	Wnt5a knockdown or overexpression, adoptive transfer and CCR2 inhibition	Human: Cross-sectional; Rat: experimental longitudinal	None	(45)
NK cells	HBV-related fibrosis (F0–F1 vs F2–F3) vs Healthy controls	KLRG1^+^ NK cells show greater TRAIL-dependent HSC killing and IFN-γ production and are enriched in less advanced fibrosis	Human liver + Peripheral blood	Human: flow cytometry + *in vitro*	HSC cytotoxicity, apoptosis, degranulation and cytokine assays; TRAIL neutralization	Cross-sectional	None	(46)
NK cells	HBV vs HBV-related cirrhosis	Activated HSC-derived TGF-β induces NK-cell emperipolesis/apoptosis and impairs HSC-directed antifibrotic activity	Human liver + peripheral blood	Human: flow cytometry + *in vitro*	TGF-β blockade, cell-contact experiments and NK functional assays	Cross-sectional + >12-month antiviral follow-up	None	(47)
NK cells	HBV-related fibrosis/cirrhosis (Laennec scores 0–2 vs 3–4)	Intrahepatic NKp46, IFN-γ and perforin decrease in advanced fibrosis despite similar NK-cell abundance	Human liver and peripheral blood	Human: flow cytometry + ex vivo	Phenotypic and functional measurements; no causal perturbation	Cross-sectional	None	(48)
NK cells	HBV-related cirrhosis vs non-cirrhotic controls	Intrahepatic NKG2D expression decreases with collagen deposition and poorer liver function	Human liver	Human: flow cytometry + histology	Phenotyping and clinicopathological correlation; no causal perturbation	Cross-sectional	None	(49)
NK cells	HBV-related cirrhosis vs Healthy controls	NK-JUNB cells accumulate, while cytotoxic programs are suppressed in cirrhosis	Human liver	Human: scRNA-seq	Subset annotation and pseudotime; no causal perturbation	Cross-sectional	None	(25)
NK cells	HBV vs HBV-related cirrhosis vs HBV-related HCC vs Healthy controls	NK-GNLY cells increase from cirrhosis to HBV-related HCC	Human liver	Human: scRNA-seq	Subset annotation and inferred cell communication	Cross-sectional	Cross-sectional HCC association	(18)
NK cells	HBV-related cirrhosis vs HBV- related HCC	Circulating NK-cell abundance and activating phenotypes, including NKp46^+^ NK cells, differ between disease groups	Peripheral blood	Human: flow cytometry	Phenotyping only	Cross-sectional	Cross-sectional HCC association	(51)
NK cells	CHB vs compensated and decompensated HBV-related cirrhosis	CXCR3 ligands are associated with recruitment of TRAIL^+^CD56bright NK cells and liver injury	Peripheral blood + serum chemokines	Human: flow cytometry + *in vitro*	PBMC–target-cell co-culture; TRAIL blockade and apoptosis assays	Cross-sectional	None	(52)
Neutrophils	CHB-related fibrosis (IshakF0-6)	Circulating NET-associated citrullinated histone H3, cell-free DNA and MPO–DNA increase with fibrosis severity	Peripheral blood	Human: circulating NET biomarkers	Circulating NET-associated biomarkers only	Cross-sectional	None	(53)
Neutrophils	Acute HBV vs CHB vs HBV-related cirrhosis	IL-1β-bearing NETs induce a profibrotic phenotype in primary human fibroblasts	Serum + peripheral blood	Human: microscopy + *in vitro*	NET fibroblast assays; DNase I and IL-1 receptor blockade	Cross-sectional	None	(54)
Neutrophils	HBV-related cirrhosis	Higher baseline NLR, a composite neutrophil–lymphocyte index, independently predicts incident HCC	Peripheral blood	Human: routine blood tests	Adjusted multivariable association; no cell-specific functional assay	Retrospective longitudinal	Pre-HCC longitudinal association	(55)
Dendritic cells	CHB vs HBV-related cirrhosis vs HBV-related HCC vs Healthy controls	DC-surface TLR2/TLR4 expression differs across HBV disease groups; DC frequency shows a non-significant downward trend	Peripheral blood	Human: flow cytometry	Phenotyping only	Cross-sectional	Cross-sectional HCC association	(60)
MDSCs	CHB vs HBV-related cirrhosis vs Healthy controls	Peripheral MDSC proportions are lower in HBV-related cirrhosis than in CHB but remain higher than in healthy controls	Peripheral blood	Human: flow cytometry	Flow-cytometric phenotyping; no functional perturbation	Cross-sectional	None	(79)
T cells	Human: HBV-related fibrosis; Mouse: ConA-induced fibrosis	Lower Treg/Th17 balance tracks injury/fibrosis; IL-17 promotes HSC activation and fibrogenesis	Human: liver + Peripheral blood; Mouse: liver	Human: flow cytometry + *in vitro*; Mouse: *in vivo*	T-cell–HSC co-culture, IL-17 blockade and CD25^+^/IL-17^+^ cell depletion in experimental fibrosis	Human: Cross-sectional; Mouse: experimental perturbation	None	(62)
T cells	HBV-related fibrosis/cirrhosis (modified Scheuer S0–S4) vs healthy controls	Activated HSCs induce IL-17A^+^IL-23R^+^RORC^+^CCR6^+^ pathogenic Th17 cells through IL-1β, IL-6 and COX–PGE_2_ signaling	Peripheral blood	Human: phenotyping + *in vitro*	HSC–T-cell co-culture and pathway perturbation	Cross-sectional	None	(63)
T cells	HBV-related cirrhosis without HCC at baseline	Peripheral Th17/Treg imbalance correlates with liver stiffness and predicts subsequent HCC	Peripheral blood	Human: flow cytometry	Flow cytometry, propensity-score matching and Cox analysis; no mechanistic perturbation	Longitudinal follow-up cohort	Pre-HCC longitudinal association	(65)
T cells	HBV vs HBV-related cirrhosis vs HBV-related HCC vs Healthy controls	Effector CD8^+^ T cells expand from infection to cirrhosis then decline after HCC emerges; Tregs and LAG3^+^ exhausted CD8^+^ T cells increase	Human liver	Human: scRNA-seq	Subset annotation and inferred cell communication	Cross-sectional	Cross-sectional HCC association	(18)
T cells	HBV-related cirrhosis vs HBV-related HCC vs Healthy controls	Circulating T-cell composition, including CD3^+^ and CD8^+^HLA-DR^+^CD38^+^ measures, differs across disease groups	Peripheral blood	Human: flow cytometry	Phenotyping and diagnostic discrimination only	Cross-sectional	Cross-sectional HCC association	(51)
T cells	CHB vs HBV-related cirrhosis vs HBV-related HCC	IFN-γ from CD161^+^CD4^+^ T cells promotes an IL-23/IL-17 circuit and HSC fibrogenesis	Human: liver + Peripheral blood	Human: flow cytometry + *in vitro*	HSC co-culture and IFN-γ/IL-23/IL-17 pathway perturbation	Cross-sectional + 52-week antiviral follow-up for virological outcome	Cross-sectional HCC context	(64)
T cells	CHB vs HBV-related cirrhosis vs HBV-related HCC vs Healthy controls	Memory-T-cell mitochondrial mass increases and the low-membrane-potential fraction decreases across disease stages	Peripheral blood	Human: flow cytometry	Phenotyping only	Cross-sectional	Cross-sectional HCC association	(66)
B cells	HBV-related fibrosis/cirrhosis (modified Scheuer S0–S4) vs healthy controls	IL-10-producing CD19^+^CD24highCD38high Bregs suppress IFN-γ/IL-17 effector T cells and promote Treg differentiation	Human: liver + Peripheral blood	Human: flow cytometry + *in vitro*	Sorted Breg–T-cell co-culture; IL-10-dependent and contact-dependent assays	Cross-sectional	None	(75)
B cells	CHB vs HBV-related cirrhosis	Peripheral CD19^+^CD24highCD38high Bregs and intrahepatic IL-10^+^PAX5^+^ Bregs increase and correlate with fibrosis stage	Human: liver + Peripheral blood	Human: flow cytometry + histology	Phenotyping and tissue correlation only; no direct functional assay	Cross-sectional	None	(76)
B cells	HBV-related cirrhosis vs Healthy controls	B-cell-IGLC1 and naïve B-cell states are depleted in HBV-related cirrhosis	Human liver	Human: scRNA-seq	Subset annotation and pseudotime; no causal perturbation	Cross-sectional	None	(25)
B cells	HBV-related cirrhosis and HBV-related HCC vs Healthy controls	Peripheral B-cell subset frequencies differ across disease groups	Peripheral blood	Human: flow cytometry	Phenotyping only	Cross-sectional	Cross-sectional HCC association	(51)
B cells	CHB vs HBV-related cirrhosis vs HBV-related HCC vs Healthy controls	Peripheral CD27^+^IgD^+^ B cells show stepwise depletion with fibrosis progression	Peripheral blood	Human: flow cytometry	Phenotyping and clinicopathological correlation; no perturbation	Cross-sectional + limited retrospective serial measurements	Cross-sectional HCC association	(77)

### 3.1 Innate immune remodeling in HBV-related fibrosis and cirrhosis

Innate immune remodeling in HBV-related fibrosis reflects a progressive redistribution of liver-resident and recruited myeloid and cytotoxic populations, with inflammatory activity varying according to disease stage and cellular context. Within the monocyte–macrophage compartment, Kupffer cells coexist with recruited monocyte-derived macrophages and multiple disease-associated states whose functions depend on anatomical localization, activation program and disease stage. Activated HSC-derived CCL2 promotes CCR2-dependent recruitment of macrophages and favors CD163^+^/CD206^+^ populations, while reciprocal macrophage–HSC signaling can sustain fibrogenic activity (41). More recent spatial profiling of human CHB liver identified CD86^+^TNF-α^+^CXCL9^+^ inflammatory macrophages adjacent to activated HSCs and collagen-rich regions, with HBV-conditioned macrophages directly enhancing HSC activation (42). Circulating myeloid remodeling is also evident before cirrhosis: single-cell profiling of CHB patients with fibrosis showed expansion of monocyte populations and identified monocyte–platelet aggregates with enhanced inflammatory and adhesive programs (43). By the cirrhotic stage, high-resolution liver profiling reveals scar-associated macrophage populations including macrophage-CD9/IL18 and macrophage-C1QA, together with altered antigen-processing programs (25). Importantly for cancer interception, macrophage remodeling continues beyond cirrhosis. Cross-stage single-cell analysis shows that macrophage-CD9/IL18 expands predominantly from HBV infection to cirrhosis, whereas macrophage-CD9/IFI6 becomes enriched from cirrhosis to HBV-HCC (18). Macrophage-associated activity is also modifiable: circulating soluble CD163 (sCD163) and soluble mannose receptor and hepatic CD163 decline during antiviral treatment, and serial intrahepatic profiling shows attenuation of inflammatory macrophage programs (28, 44). Taken together, the macrophage axis already fulfils three elements relevant to interception—association with fibrotic severity, antiviral-treatment responsiveness, and a stage-associated link to HCC. Its HCC linkage is currently supported mainly by cross-sectional disease-stage reconstruction, with prospective incident HCC prediction requiring further evidence; macrophages can therefore be considered an HCC-linked interception candidate at the stage-association level, alongside their established role in fibrosis biology. Comparative single-cell analysis further showed expansion of macrophage-PLCG2 and contraction of macrophage-CD5L in HBV-related cirrhosis, while complementary human and experimental work implicated macrophage Wnt5a–FZD2 signaling in hepatic progenitor-cell fate and fibrogenesis (22, 45).

NK cells represent another biologically coherent interception axis because their function directly spans both fibrosis control and tumor immunosurveillance. In CHB, killer cell lectin-like receptor G1 (KLRG1)^+^ NK cells exhibit enhanced TNF-related apoptosis-inducing ligand (TRAIL)-dependent killing of activated HSCs and greater interferon-γ (IFN-γ) production and are enriched in patients with less advanced fibrosis (46). With progression toward advanced fibrosis and cirrhosis, this antifibrotic capacity is progressively impaired: activated HSC-derived transforming growth factor-β (TGF-β) promotes NK-cell emperipolesis and apoptosis, and reductions in natural cytotoxicity receptor NKp46, natural killer group 2 member D (NKG2D), perforin and IFN-γ accompany more advanced disease (47–50). Single-cell analysis of HBV cirrhosis further demonstrates expansion of dysfunctional NK states and broad suppression of NK-cell cytotoxic programs (25). The transition to established HCC is accompanied by further redistribution of NK functional states: NK-GNLY cells expand from cirrhosis to HCC, and peripheral comparisons between HBV cirrhosis and treatment-naïve HBV-HCC identify differences in activating NK phenotypes, including NKp46^+^ NK cells (18, 51). These findings link the loss and reconfiguration of NK cytotoxic surveillance to disease progression from fibrosis and cirrhosis to HCC. Moreover, experimental TGF-β blockade can partially restore HSC-directed NK activity, providing proof that this state is biologically reversible (47). The disease-side NK evidence therefore supports a biologically modifiable trajectory with cross-sectional HCC linkage. What is not yet demonstrated is that restoration of a defined antifibrotic NK state in patients subsequently lowers HCC incidence. CXCR3-ligand-associated recruitment of TRAIL^+^CD56bright NK cells in compensated and decompensated HBV cirrhosis further indicates that NK-cell accumulation may accompany liver injury, underscoring the need to distinguish antifibrotic cytotoxic states from inflammation-associated recruitment (52).

Neutrophils contribute through a distinct inflammatory program centered on neutrophil extracellular traps (NETs). Circulating citrullinated histone H3, cell-free DNA and myeloperoxidase–DNA (MPO–DNA) complexes increase with Ishak fibrosis stage in untreated CHB, and sera from HBV-related disease can induce IL-1β-bearing NETs that promote profibrotic activation of fibroblasts (53, 54). This provides a cell-resolved fibrosis mechanism, but the HCC-related evidence is currently measured at a substantially lower resolution. In HBV cirrhosis cohorts, the neutrophil-to-lymphocyte ratio (NLR) has been associated with subsequent HCC occurrence, including long-term clinical cohorts incorporated in this review (55). NLR therefore provides a genuine longitudinal HCC-linked inflammatory signal, but it cannot establish whether the association derives from NET formation, neutrophil expansion, lymphocyte depletion or another systemic process. Consequently, neutrophils should not yet be described as having a complete NET-to-HCC trajectory: the fibrosis arm is cell-resolved, whereas the cancer-interception arm remains a composite proxy.

Dendritic cells (DCs) and myeloid-derived suppressor cells (MDSCs) primarily influence HBV disease through regulation of antiviral immunity and immune tolerance rather than through a well-defined fibrosis-specific trajectory. Simultaneous profiling of circulating and intrahepatic conventional dendritic cell type 1 and type 2 (cDC1 and cDC2) populations and plasmacytoid dendritic cell (pDC) populations demonstrates altered distribution, co-stimulatory molecules, Toll-like receptor (TLR) expression and cytokine responsiveness in chronic HBV infection (56). These abnormalities are relevant because defective antigen presentation and interferon responses can weaken downstream T- and NK-cell surveillance; however, the same DC phenotypes have not been reproducibly traced from histological fibrosis through cirrhosis to subsequent HCC. DCs therefore contribute to the HBV-specific immune context of interception, but current evidence does not justify classifying a defined DC population as an HCC-linked interception trajectory. MDSCs show a clearer functional suppressive phenotype. Arginase-expressing granulocytic MDSCs accumulate in blood and liver during persistent HBV infection, deplete L-arginine and suppress antiviral T-cell function, while HSCs facilitate their expansion (57, 58). Additional work demonstrates distinct MDSC phenotypes across chronic HBV phases and persistent MDSC abnormalities despite tenofovir therapy (59). These observations establish an intervention-relevant immunosuppressive program, but available studies link MDSCs more consistently to viral persistence and immunopathology than to histologically defined fibrosis progression or future HCC. Accordingly, MDSCs remain a regulatory candidate relevant to the biological pre-HCC environment, but do not currently constitute a cell-resolved HCC-linked trajectory in the same sense as T-cell, macrophage or NK-cell changes. A second cross-sectional study found that peripheral dendritic-cell frequency and surface TLR2/TLR4 expression varied across HBV-carrier, CHB, cirrhosis and HBV-HCC groups (60); this broad stage association does not by itself define a fibrosis-specific dendritic-cell trajectory.

### 3.2 Adaptive immune remodeling in fibrosis and HCC

#### 3.2.1 T-cell remodeling

T-cell remodeling in HBV-related fibrosis is defined less by the total numbers of CD4^+^ or CD8^+^ T cells than by redistribution among inflammatory, regulatory, effector and exhausted states. Chronic HBV exposure progressively constrains virus-specific CD8^+^ T-cell function, whereas antigen-nonspecific or bystander T-cell responses can contribute to hepatocyte injury. Serial liver fine-needle aspirates during antiviral-treatment initiation identified polyclonal CXCR6^+^CD8^+^ T cells expressing IFNG and FASLG during active hepatitis; this population contracted as biochemical injury improved, directly demonstrating reversibility of an intrahepatic inflammatory CD8^+^ program (29, 61). Within the CD4^+^ compartment, T helper 17 (Th17) and regulatory T (Treg) cells provide a particularly informative fibrosis axis. In biopsy-characterized HBV fibrosis, both populations are altered, and the Treg/Th17 balance correlates with histological injury and fibrosis; IL-17 directly promotes HSC activation, whereas depletion or functional manipulation of these populations alters experimental fibrogenesis (62). Activated HSCs can, in turn, induce more pathogenic IL-17A^+^IL-23R^+^RORC^+^CCR6^+^ Th17 cells through IL-1β, IL-6 and cyclooxygenase–prostaglandin E_2_ (COX–PGE_2_) signaling, establishing a functionally validated feedback between adaptive immunity and fibrotic stroma (63). These findings establish Th17/Treg remodeling not merely as a circulating biomarker but as a biologically active and modifiable component of HBV fibrosis. An additional CD4^+^ program links IFN-γ-producing CD161^+^CD4^+^ T cells to an IL-23/IL-17 circuit that promotes hepatic stellate-cell fibrogenesis (64).

The T-cell axis also contains the clearest current human evidence that a fibrosis-associated immune change can extend into incident HCC risk. In a longitudinal HBV-cirrhosis cohort, the peripheral Th17/Treg ratio correlated with liver stiffness and was independently associated with subsequent HCC occurrence (65). Although peripheral blood cannot be assumed to reproduce intrahepatic immunity, this study directly places a pre-existing functional T-cell imbalance before the clinical HCC endpoint and therefore fulfils the definition of an HCC-linked interception candidate. Cross-stage liver single-cell data provide complementary cellular resolution: effector CD8^+^ T-cell populations expand from HBV infection to cirrhosis but decline after HCC emerges, while Treg cells and LAG3^+^ exhausted CD8^+^ T cells increase from cirrhosis to HCC (18). Peripheral immune profiling independently identifies increased activated CD8^+^HLA-DR^+^CD38^+^ states and other T-cell phenotypes that discriminate HBV-HCC from HBV cirrhosis (51). These datasets converge on a transition from inflammatory/effector T-cell activity during fibrotic disease toward increasing regulatory and exhausted features around HCC emergence. Unlike the Th17/Treg cohort, however, these single-cell and case-control patterns are stage associations rather than prospective trajectories. The T-cell compartment therefore contains both longitudinal and cross-sectional cancer-interception evidence, making it the most clearly developed HCC-linked immune-cell axis within the evidence synthesized here, without implying that every observed T-cell subtype is itself an interception target. Cross-sectional mitochondrial profiling also identified stage-associated changes in memory-T-cell mitochondrial mass and membrane-potential distributions across CHB, cirrhosis and HBV-HCC (66), providing a metabolic correlate rather than a prospective trajectory.

#### 3.2.2 IL-17 family in HCC

Beyond its profibrotic role, IL-17 also shapes the microenvironment of established HCC. In resected HCC, higher intratumoral IL-17-producing-cell density was associated with poorer overall and disease-free survival, while intratumoral Th17 frequency correlated with microvessel density (67). Experimental studies further link IL-17A to STAT3-dependent induction of fibroblast activation protein (FAP) in HSCs, supporting stromal activation and tumor growth (68).

The effects of IL-17A on immunotherapy are context-dependent. In experimental HCC, pathogenic Th17-derived IL-17A promoted tumor-cell programmed death-ligand 1 (PD-L1) expression and resistance to PD-L1 blockade (69). IL-17/IL-17RA signaling also induced PD-L1 through nuclear factor kappa B (NF-κB) activation (70). Conversely, histone deacetylase 6 (HDAC6)-deficient Th17 cells enhanced IL-17A-dependent antitumor responses; IL-17A blockade partially attenuated the benefit of combining HDAC6-depleted T cells with checkpoint inhibition (71).

Functional heterogeneity also extends across the IL-17 family (72). IL-25 (IL-17E) promoted macrophage alternative activation and CXCL10-dependent HCC-cell migration and invasion (73), whereas IL-17B inhibited proliferation in HepG2 and Hep3B cells (74). These findings concern established-HCC biology and treatment; their ligand-, cell-state- and treatment-dependent effects indicate that CHM-associated reductions in Th17/IL-17 readouts alone cannot establish restored antitumor immunity or reduced HCC incidence.

#### 3.2.3 B-cell remodeling

B-cell remodeling follows a different and less continuous pattern. IL-10-producing CD19^+^CD24^hi^CD38^hi^ regulatory B (Breg) cells increase with advanced histological HBV fibrosis and suppress IFN-γ- and IL-17-producing effector T cells while enhancing Treg differentiation through IL-10-dependent and cell-contact mechanisms (75, 76). This establishes a functional fibrosis-associated regulatory B-cell state. HBV cirrhosis is associated with loss of an IGLC1-enriched B-cell cluster and depletion of naïve B-cell states, while peripheral B-cell frequency is lower in HBV-HCC than in HBV cirrhosis (25, 51). These observations clearly show that B-cell composition changes along the fibrosis–HCC continuum, but the currently reported signals involve different B-cell populations and cannot yet be assembled into one continuous Breg-to-HCC trajectory. This distinction is especially relevant when later evaluating CHM studies, because a change in total B-cell abundance would not demonstrate reversal of a regulatory B-cell program. Consistent with this compositional remodeling, peripheral CD27^+^IgD^+^ B cells show stepwise depletion across chronic HBV, cirrhosis and cirrhosis with HCC (77, 78).

### 3.3 Which HBV immune-cell trajectories currently support cancer interception?

Taken together, the disease-side evidence supports a clear hierarchy across immune-cell populations. Accordingly, the Th17/Treg axis reaches the HCC-linked candidate level with longitudinal human evidence; macrophage and NK trajectories reach that level mainly through cross-stage and functional evidence; neutrophil/NLR evidence remains proxy-level; B-cell evidence is incomplete; and DC/MDSC evidence remains context-level. These findings identify candidate immune targets, but whether modifying them reduces subsequent HCC incidence remains unproven.

## 4 Chinese herbal medicine modulation of HBV-associated immune trajectories and cancer interception

The included CHM interventions comprise multi-herb formulations and constituent-based preparations. Their reported composition, source, study setting, dose and regimen, and available preparation or quality-control information are summarized in **Supplementary Table S2**. Constituents, extracts and multi-herb preparations were treated as distinct exposures, with constituent effects interpreted at the constituent level and whole-preparation effects at the formulation level.

A 2026 systematic review of 25 randomized controlled trials involving 2,585 patients with HBV-related fibrosis or cirrhosis showed that CHM combined with nucleos(t)ide analogs was associated with higher circulating CD3^+^ and CD4^+^ T-cell measures, a higher CD4^+^/CD8^+^ ratio and increased NK-cell-related indices, together with lower CD8^+^ T-cell measures, inflammatory mediators and fibrosis-related markers (31, 80–82). These findings indicate that immune remodeling is a recurring component of the antifibrotic effects observed with CHM, although the depth of immune characterization varies substantially across studies, ranging from broad circulating-cell measurements to functional subsets, receptor phenotypes, liver-tissue analyses and cell-specific mechanistic experiments. Ruangan Granule plus entecavir was likewise associated with histological regression and fewer HCC events during follow-up, but without serial immune-cell profiling; it therefore functions as a clinical-outcome anchor rather than cell-specific immunomechanistic evidence (33).

To characterize the overall evidence landscape, immune-cell categories were evaluated across six dimensions of evidence maturity (**Figure 3**, **Table 5**). In parallel, a separate four-tier framework was applied at the level of individual intervention–immune mechanism pairs to define the highest cancer-interception inference supported by each pair (**Figure 4**, **Table 6**). **Supplementary Table S3** summarizes the study designs, HCC outcomes and confounding control of the clinical outcome reports included in **Table 6**. The combination-specific evidence-maturity profiles and interception tiers are summarized in **Figure 5**.

**Figure 3:**
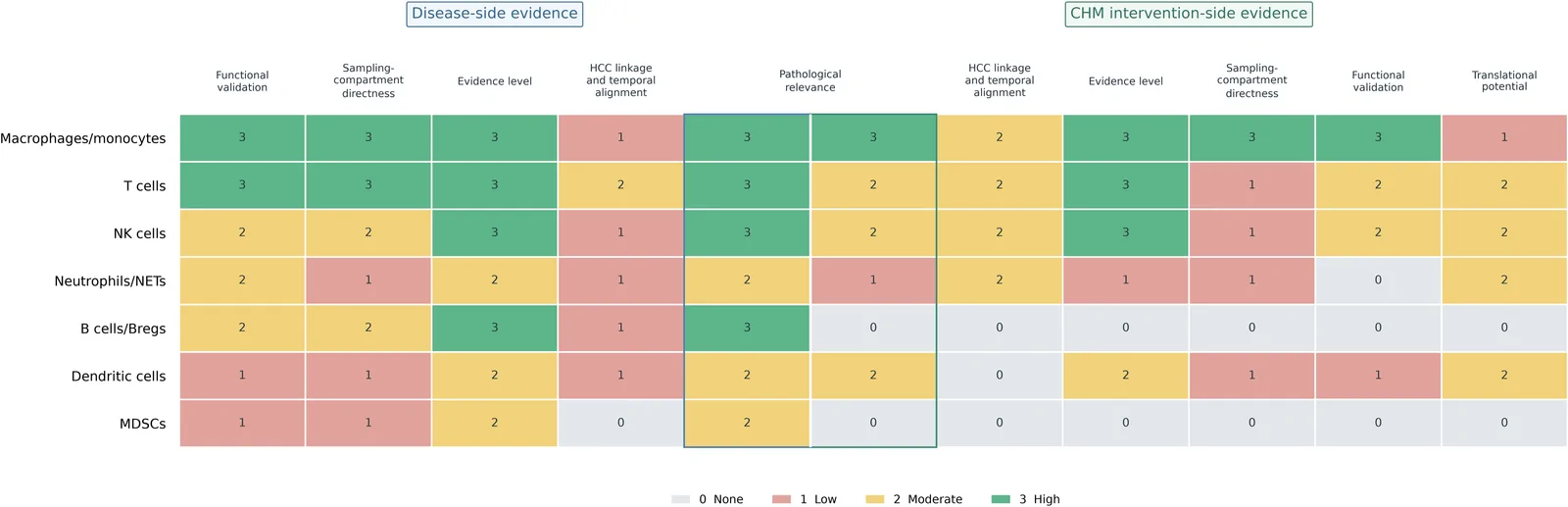
Evidence-maturity profiles of disease-side and CHM intervention evidence across immune-cell populations. The heatmap summarizes disease-side and CHM intervention-side evidence across immune-cell populations using the ratings in **Table 5**. The two evidence streams are scored independently, including pathological relevance. From the central pathological-relevance columns outwards, dimensions follow the D1–D5 sequence on the disease side and D1–D6 on the CHM side; translational potential applies only to CHM intervention evidence. Scores range from 0 to 3, indicating no qualifying evidence, low, moderate and high evidence maturity, respectively. Grey denotes evidence gaps. (CHM, Chinese herbal medicine; HCC, hepatocellular carcinoma; NETs, neutrophil extracellular traps; Bregs, regulatory B cells; MDSCs, myeloid-derived suppressor cells).

**Table 5:** Evidence-maturity ratings for disease-side and CHM intervention-side immune-cell evidence.

Disease-side five-dimensional evidence ratings					CHM intervention-side six-dimensional evidence ratings		
Immune-cell population	Dimension	Score (0–3)	Rationale	Ref.	Score (0–3)	Rationale	Ref.
Macrophages/monocytes	Pathological relevance	3	Multiple human HBV liver studies directly link recruited and resident macrophage states to activated HSCs, collagen-rich niches, fibrosis severity and cirrhosis.	(25, 41–45)	3	Paired human HBV liver biopsies show reduced NLRP3 staining after FZHY plus entecavir; complementary macrophage–HSC experiments link the pathway to fibrogenic responses.	(39)
Macrophages/monocytes	HCC linkage and temporal alignment	1	Macrophage-state shifts are reported across cirrhosis and established HBV-HCC only; no macrophage trajectory has prospectively predicted incident HCC.	(18)	2	FZHY has macrophage/NLRP3 functional evidence and separate longitudinal HCC outcome studies, but immune mediation was not measured in the outcome cohorts.	(35, 39, 40, 93)
Macrophages/monocytes	Evidence level	3	The evidence includes several human HBV tissue studies, longitudinal liver sampling and complementary animal/cell perturbation.	(22, 25, 41–45)	3	Human HBV fibrotic liver, HBV-mouse experiments and macrophage/HSC assays directly evaluate CHM-responsive macrophage mechanisms.	(39, 83)
Macrophages/monocytes	Sampling-compartment directness	3	Spatial human-liver profiling and paired biopsies provide direct and repeated liver anchoring.	(22, 25, 41–44)	3	Paired pre/post human HBV liver biopsies assess NLRP3 under Fuzheng Huayu treatment; human staining is not macrophage-specific.	(39)
Macrophages/monocytes	Functional validation	3	Macrophage–HSC co-culture, CCR2 antagonism, Wnt5a manipulation and adoptive-transfer experiments demonstrate functional dependency.	(41–43, 45)	3	EGCG-related autophagy inhibition and ATG5 knockdown reverse HMGB1/inflammasome and fibrotic readouts, supporting dependency of the tested pathway.	(83)
Macrophages/monocytes	Translational potential				1	Human evidence shows hepatic NLRP3 changes; fibrosis improvement is established only preclinically in the verified evidence.	(39)
T cells	Pathological relevance	3	Human liver/blood studies and HSC assays directly link Th17/Treg imbalance and IL-23/IL-17 signaling to HBV fibrosis.	(62–65)	2	CHM-responsive peripheral T-cell readouts accompany fibrosis-marker changes, and Fuzheng Huayu T-cell–HSC assays show functional effects; direct human intrahepatic validation is lacking.	(84, 86–90)
T cells	HCC linkage and temporal alignment	2	A pre-HCC longitudinal HBV-cirrhosis cohort links Th17/Treg imbalance to subsequent HCC, supported by cross-stage HCC comparisons.	(18, 51, 65)	2	BJRG and FZHY combine T-cell studies with separate longitudinal HCC outcome evidence; the immune and outcome observations are not one mediation cohort.	(32, 34, 35, 40, 87, 88, 90, 93)
T cells	Evidence level	3	Multiple human HBV cohorts are complemented by mouse models and cell-based experiments.	(62–66)	3	Human FZHY intervention evidence is complemented by sorted T-cell–HSC experiments and animal studies.	(90)
T cells	Sampling-compartment directness	3	Human liver analyses spatially localize CD161^+^CD4^+^ T cells in relation to activated HSCs, directly anchoring the mechanism to the liver.	(62, 64)	1	Immune measurements are predominantly peripheral; no paired pre/post intrahepatic T-cell mapping is available.	(84–90)
T cells	Functional validation	3	IL-17 blockade, CD25+/IL-17+ cell depletion and pathway perturbation support causal function.	(62–64)	2	Sorted T-cell–HSC co-culture demonstrates a functional consequence, but subset-specific *in vivo* causal tests are lacking.	(90)
T cells	Translational potential				2	FZHY study assesses peripheral T-cell readouts alongside liver stiffness and histological fibrosis outcomes, without linking immune changes to subsequent HCC.	(90)
NK cells	Pathological relevance	3	Direct human HBV liver studies link NK-cell phenotype and HSC-directed cytotoxic function to fibrosis severity and cirrhosis.	(25, 46–49)	2	FZHY NK-cell–HSC assays provide human functional evidence, while matrine changes peripheral NK phenotype in HBV cirrhosis; direct human intrahepatic validation is lacking.	(90, 91)
NK cells	HCC linkage and temporal alignment	1	HCC linkage is limited to cross-sectional comparisons involving established HBV-HCC; no prospective incident-HCC association is demonstrated.	(18, 51)	2	FZHY has NK-cell functional evidence and separate longitudinal HCC outcome evidence, but NK mediation is not measured in the outcome studies.	(35, 40, 90, 93)
NK cells	Evidence level	3	Several human HBV studies combine liver phenotyping with HSC/NK functional experiments.	(25, 46–49, 52)	3	Human HBV studies of Fuzheng Huayu and matrine are supplemented by mouse and HSC co-culture work.	(90, 91)
NK cells	Sampling-compartment directness	2	Direct or paired liver–blood measurements are available, but serial pre/post liver sampling is absent.	(46–49)	1	The immune readouts are peripheral; direct pre/post intrahepatic NK measurement is absent.	(90, 91)
NK cells	Functional validation	2	HSC-killing assays and TGF-β blockade show function, without *in vivo* depletion, genetic rescue.	(46, 47)	2	Sorted NK-cell–HSC co-culture demonstrates functional effects on HSC activation and collagen readouts, without NK-specific *in vivo* blockade or rescue.	(90)
NK cells	Translational potential				2	FZHY study assesses peripheral NK-cell readouts alongside liver stiffness and histological fibrosis outcomes, without linking immune changes to subsequent HCC.	(90)
Neutrophils/NETs	Pathological relevance	2	Human HBV studies associate circulating NET markers with fibrosis and show NET-containing serum can induce a profibrotic fibroblast phenotype, but liver anchoring is lacking.	(53, 54)	1	FZHY was associated with reductions in peripheral NLR and serum fibrosis markers in HBV-related cirrhosis; NLR provides indirect, non-cell-specific evidence of immune–fibrotic relevance.	(92)
Neutrophils/NETs	HCC linkage and temporal alignment	1	A longitudinal incident HCC association exists for baseline NLR, a composite proxy, rather than for the defined NET/neutrophil state.	(55)	2	Separate longitudinal studies associate the FZHY intervention with lower subsequent HCC incidence.	(35, 40, 90, 93)
Neutrophils/NETs	Evidence level	2	The evidence is limited to human biomarker/ex vivo studies and one longitudinal cohort without liver-resolved validation.	(53–55)	1	Evidence relies on peripheral NLR as an indirect immune proxy, without direct assessment of a neutrophil-specific mechanism.	(92)
Neutrophils/NETs	Sampling-compartment directness	1	Measurements are confined to peripheral blood or serum.	(53–55)	1	Only peripheral blood and clinical outcomes are measured.	(92)
Neutrophils/NETs	Functional validation	2	NET–fibroblast assays demonstrate functional effects, but *in vivo* causal validation is lacking.	(54)	0	No functional perturbation or causal validation	—
Neutrophils/NETs	Translational potential				2	FZHY was associated with reductions in serum fibrosis markers in patients with HBV-related cirrhosis.	(92)
B cells/Bregs	Pathological relevance	3	Human HBV studies identify intrahepatic Breg accumulation and stepwise B-cell-subset depletion in relation to fibrosis progression and cirrhosis.	(25, 75–77)	0	No qualifying CHM–B-cell mechanism is retained; disease-side pathological relevance is not transferred.	—
B cells/Bregs	HCC linkage and temporal alignment	1	B-cell HCC evidence is cross-sectional; no B-cell trajectory has been linked prospectively to incident HCC.	(18, 51, 77)	0	No retained CHM intervention–B-cell mechanism is linked to HCC outcome.	—
B cells/Bregs	Evidence level	3	Multiple human HBV cohorts are supported by sorted Breg–T-cell functional experiments.	(75–77)	0	No direct CHM–B-cell intervention study is retained in HBV fibrosis/cirrhosis.	—
B cells/Bregs	Sampling-compartment directness	2	Both peripheral and intrahepatic B-cell measurements are available, without serial or spatial pre/post mapping.	(25, 76)	0	No intervention-linked B-cell compartment was measured.	—
B cells/Bregs	Functional validation	2	Sorted Breg co-culture and IL-10/contact-dependence assays demonstrate immune function but not disease-endpoint causality.	(75)	0	No CHM-related B-cell functional validation was retained.	—
B cells/Bregs	Translational potential				0	No qualifying CHM–B-cell combination is retained to which intervention-level clinical outcomes can be assigned.	—
Dendritic cells	Pathological relevance	2	Human HBV study shows stage-associated changes in peripheral DC phenotype, but the link to fibrosis-specific pathology is limited.	(60)	2	Kushenin-associated peripheral DC and CD80/CD86 changes accompany lower serum fibrosis markers in CHB, without liver-resolved validation.	(86)
Dendritic cells	HCC linkage and temporal alignment	1	HCC evidence is limited to a cross-sectional comparison of peripheral DC frequency and TLR expression.	(60)	0	No retained CHM intervention–DC mechanism is linked to HCC outcome.	—
Dendritic cells	Evidence level	2	Small human evidence assesses peripheral DC phenotype without complementary mechanistic validation.	(60)	2	A comparative human CHB study measures peripheral DC phenotype under kushenin, without complementary mechanistic validation.	(86)
Dendritic cells	Sampling-compartment directness	1	DC measurements are confined to peripheral blood.	(60)	1	DC and CD80/CD86 measurements are confined to peripheral blood.	(86)
Dendritic cells	Functional validation	1	DC frequency and TLR2/TLR4 expression are measured without functional perturbation.	(60)	1	mDC/pDC and CD80/CD86 changes provide phenotypic evidence only; antigen-presentation or causal assays were not performed.	(86)
Dendritic cells	Translational potential				2	Kushenin study reports lower serum PC-III and C-IV, without longitudinal incident HCC outcomes.	(86)
MDSCs	Pathological relevance	2	Peripheral MDSC proportions differ across HBV disease stages, but fibrosis-specific mechanistic evidence is limited.	(79)	0	No qualifying CHM–MDSC mechanism is retained; disease-side pathological relevance is not transferred.	—
MDSCs	HCC linkage and temporal alignment	0	No HCC linkage is retained for MDSCs in the fixed evidence table.	—	0	No retained CHM intervention–MDSC mechanism is linked to HCC outcome.	—
MDSCs	Evidence level	2	A human cross-sectional study directly measures circulating MDSC proportions without complementary mechanistic validation.	(79)	0	No direct CHM–MDSC intervention study is retained in HBV fibrosis/cirrhosis.	—
MDSCs	Sampling-compartment directness	1	MDSC measurements are confined to peripheral blood.	(79)	0	No intervention-linked MDSC compartment was measured.	—
MDSCs	Functional validation	1	Flow-cytometric phenotyping only; no functional suppression or perturbation assays were performed.	(79)	0	No CHM-related MDSC functional validation was retained.	—
MDSCs	Translational potential				0	No qualifying CHM–MDSC combination is retained to which intervention-level clinical outcomes can be assigned.	—

Disease-side evidence is rated across five applicable dimensions; the CHM side adds translational potential. The two evidence streams are scored independently. At the immune-cell-category level, each rating reflects the highest fully supported level for that dimension within the retained evidence; different studies or CHM–immune combinations may support different dimensions. A CHM-side score of 0 denotes no qualifying retained evidence for that category and dimension. (BJRG, Biejia-Ruangan; FZHY, Fuzheng Huayu; CHM, Chinese herbal medicine; HBV, hepatitis B virus; HCC, hepatocellular carcinoma; HSC, hepatic stellate cell; NET, neutrophil extracellular trap; NLR, neutrophil-to-lymphocyte ratio; Breg, regulatory B cell; DC, dendritic cell; MDSC, myeloid-derived suppressor cell; PC-III, type III procollagen; C-IV, type IV collagen).

**Figure 4:**
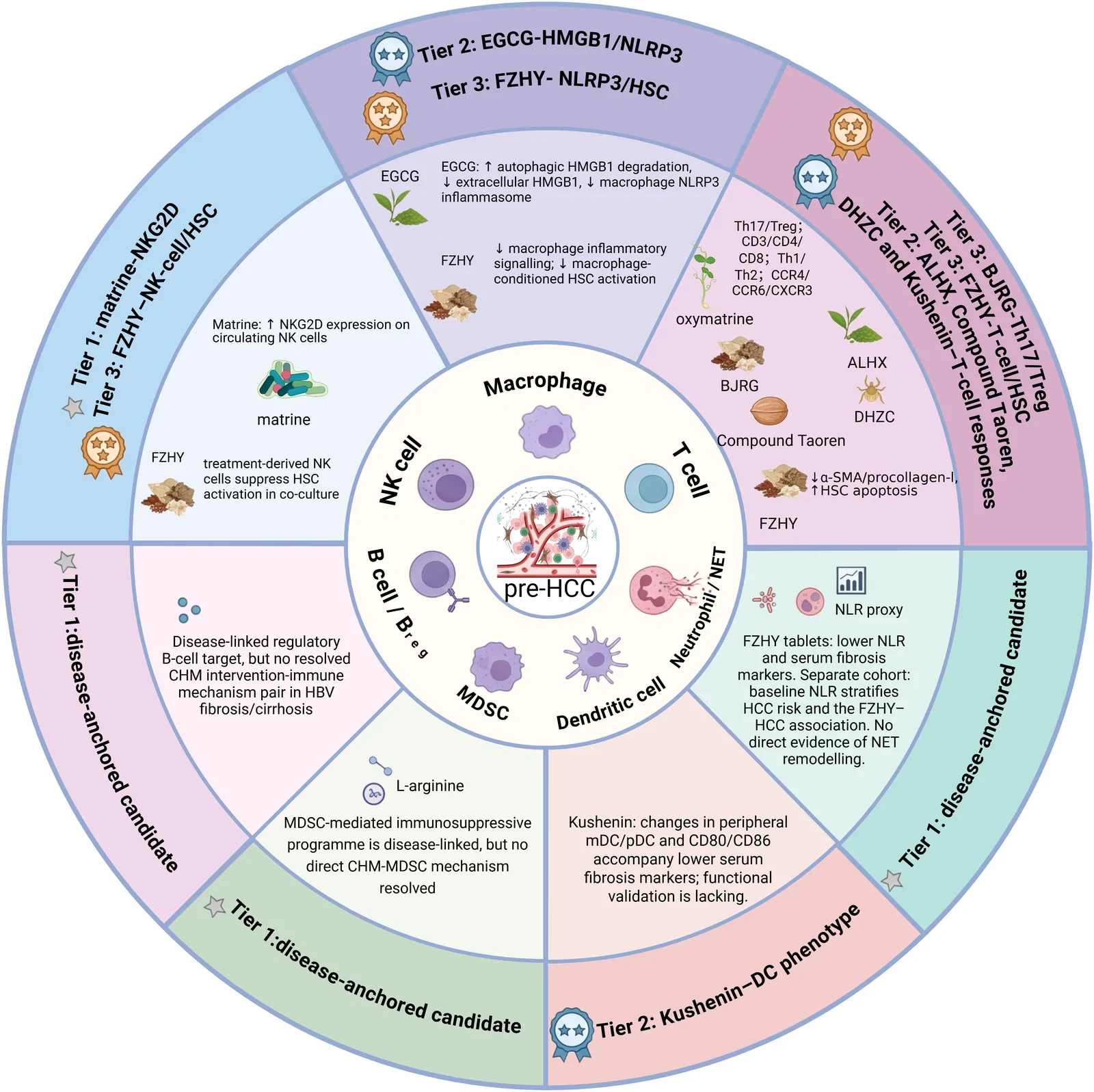
Mapping CHM-associated immune evidence onto the four-tier cancer-interception framework. Colored sectors represent immune-cell categories, with inner labels summarizing reported intervention-associated changes or evidence gaps and outer labels indicating the highest supported tier. Tier 1 labels for B cells, MDSCs and neutrophils/NETs denote disease-side candidate targets rather than established CHM-responsive mechanisms; CHM-associated NLR changes remain non-cell-specific proxy evidence. The matrine–NKG2D pair remains Tier 1 because accompanying fibrosis-related improvement was not established in the assessed evidence. Tier 2 indicates immune-target changes accompanied by improvement in fibrosis-related measures. Tier 3 reflects convergence with separate HCC outcome evidence for the same intervention, not demonstrated immune mediation of lower HCC incidence. No combination meets Tier 4 criteria. Upward and downward arrows denote reported increases and decreases, respectively. (ALHX, Anluo Huaxian; BJRG, Biejia-Ruangan; Breg, regulatory B cell; CCR, C–C chemokine receptor; CD, cluster of differentiation; CHM, Chinese herbal medicine; CXCR, C–X–C chemokine receptor; DC, dendritic cell; DHZC, Dahuang Zhechong; EGCG, epigallocatechin-3-gallate; FZHY, Fuzheng Huayu; HBV, hepatitis B virus; HCC, hepatocellular carcinoma; HMGB1, high-mobility group box 1; HSC, hepatic stellate cell; MDSC, myeloid-derived suppressor cell; mDC, myeloid dendritic cell; NET, neutrophil extracellular trap; NK, natural killer; NKG2D, natural killer group 2 member D; NLR, neutrophil-to-lymphocyte ratio; NLRP3, NLR family pyrin domain-containing 3; pDC, plasmacytoid dendritic cell; Th, T helper; Treg, regulatory T cell; α-SMA, alpha-smooth muscle actin). Created in BioRender. yang, J. (2026) https://BioRender.com/f9n8sit.

**Table 6:** CHM interventions targeting immune mechanisms in HBV-related fibrosis, cirrhosis and the pre-HCC period.

Immune-cell population	CHM intervention and exposure class	HBV disease stage/model	Main reported immune effect/pathway	Sampling compartment	Evidence system	Functional validation	Temporal design	HCC linkage	Ref.
Macrophages/monocytes	Epigallocatechin-3-gallate (purified constituent)	Mouse: rcccDNA chronic-HBV fibrosis	Hepatocyte autophagy lowers extracellular HMGB1 and macrophage NLRP3 inflammasome activation	Mouse liver	Mouse: *in vivo*; *in vitro* assays	Autophagy blockade; inflammasome/HMGB1 assays	Experimental longitudinal	None	(83)
Macrophages/monocytes	Fuzheng Huayu recipe + entecavir (standardized formula)	Human: HBV-related fibrosis; Mouse: DDC-induced fibrosis	Reduced macrophage NLRP3 inflammasome activation and cytokine release; attenuated macrophage-conditioned HSC activation	Human liver + mouse liver	Human: histology; Mouse: *in vivo* + *in vitro*	Macrophage inflammasome assays and macrophage-conditioned HSC experiments	Human: 48-week treatment follow-up; Mouse: experimental longitudinal	None; independent same-intervention incident HCC evidence available	(39)
T cells and NK cells	Fuzheng Huayu capsule + NAs (standardized formula)	Human: HBV-related fibrosis/cirrhosis; Mouse: ConA-induced fibrosis	NK-cell proportion increases; patient lymphocytes suppress HSC activation and ECM production	Human: Peripheral blood; Mouse: peripheral blood + liver	Human: flow cytometry + *in vitro*; Mouse: *in vivo* + *in vitro*	Lymphocyte-HSC co-culture	Human: Prospective comparative, 12 ± 2 months; Mouse: experimental longitudinal	None; independent same-intervention incident HCC evidence available	(90)
T cells	Biejia-Ruangan tablet + entecavir (standardized formula)	CHB with serum fibrosis assessment	Th1 increases; Th2, Th17 and Treg cells and inflammatory/collagen-turnover markers decrease	Peripheral blood	Human: flow cytometry	Phenotyping only; no functional perturbation	Prospective comparative; 24 weeks assessment	None; independent same-intervention incident HCC evidence available	(87)
T cells	Biejia-Ruangan tablet + entecavir (standardized formula)	HBV-related fibrosis	Th17-cell CCR4, CCR6 and CXCR3 expression decreases with fibrosis markers	Peripheral blood	Human: flow cytometry	Phenotyping only; no functional perturbation	Prospective comparative; 48 weeks	None; independent same-intervention incident HCC evidence available	(88)
T cells	Anluo Huaxian Pill + entecavir (standardized formula)	HBV-related cirrhosis	IL-2 and IFN-γ increase; IL-4 and IL-10 decrease with fibrosis markers	Serum	Human: cytokine measurements	Cytokine measurements only; no functional perturbation	Prospective comparative; 3 months	None	(85)
T cells	Compound Taoren capsule + entecavir (standardized formula)	HBV-related fibrosis	Treg/Th17 ratio, IL-6, TNF-α and MMP-2 decrease	Peripheral blood	Human: phenotyping + cytokine measurements	Phenotyping and cytokine measurements; no functional perturbation	Prospective randomized comparative; 6 months	None	(89)
T cells	Dahuang Zhechong capsule + entecavir (standardized formula)	HBV-related cirrhosis	CD3^+^, CD4^+^ and CD4^+^/CD8^+^ increase; CD8^+^ decreases	Peripheral blood	Human: flow cytometry	Phenotyping only; no functional perturbation	Prospective randomized; 24 weeks	None	(84)
T cells and dendritic cells	Oxymatrine/Kushenin + entecavir (defined constituent preparation)	CHB with serum fibrosis assessment	Th1 and IFN-γ decrease; Th2, mDC/pDC and CD80/CD86 expression increase, with lower serum fibrosis markers.	Peripheral blood	Human: phenotyping + cytokine measurements	Phenotyping and cytokine measurements; no functional perturbation	Randomized comparative; 3 and 6months	None	(86)
NK cells	Matrine + Nas in 43/50 patients (defined constituent)	HBV-related cirrhosis	NKG2D increases; NKG2A and NKp46 show no consistent change	Peripheral blood	Human: flow cytometry	Receptor phenotyping only; no functional perturbation	Prospective single-arm; 1 month	None	(91)
Clinical outcome anchor	Biejia-Ruangan tablet + entecavir (standardized formula)	HBV-related advanced fibrosis/cirrhosis	Greater histological fibrosis and cirrhosis regression	Human liver	Human: histology	Histology assessment; no immune-mediation test	Randomized trial; 72 weeks	None	(32)
Clinical outcome anchor	Biejia-Ruangan tablet + entecavir (standardized formula)	HBV-related advanced fibrosis/cirrhosis; no baseline HCC	Lower 7-year HCC incidence	Not applicable	Human: clinical follow-up	No immune-mediation analysis	Trial-derived prospective follow-up; median 7 years	Pre-HCC longitudinal association	(34)
Clinical outcome anchor	Fuzheng Huayu capsule + NAs (standardized formula)	HBV-related cirrhosis; no baseline HCC	Baseline NLR stratifies the observed association with lower 5-year HCC incidence; no immune-state change measured	Peripheral blood	Human: clinical follow-up + baseline NLR	No immune-mediation analysis	Retrospective longitudinal; 5 years	Pre-HCC longitudinal association	(93)
Neutrophils (NLR proxy)	Fuzheng Huayu tablet + entecavir (standardized formula)	Compensated HBV-related cirrhosis	Peripheral NLR and serum fibrosis markers decrease; no neutrophil-specific mechanism established.	Peripheral blood + serum	Human: routine blood tests + serum biomarkers	NLR measurement only; no functional perturbation	Randomized comparative; 24 weeks	None	(92)
Clinical outcome anchor	Fuzheng Huayu tablet + entecavir (standardized formula)	Compensated HBV-related cirrhosis	Lower 5-year HCC incidence after matching	Not applicable	Human: clinical follow-up	No immune-mediation analysis	Retrospective longitudinal; median 39 months; 5-year estimates	Pre-HCC longitudinal association	(35)
Clinical outcome anchor	Fuzheng Huayu capsule + NAs (standardized formula)	HBV-related cirrhosis	Longer exposure associated with lower HCC incidence	Not applicable	Human: clinical follow-up	No immune-mediation analysis	Retrospective longitudinal; 5 years	Pre-HCC longitudinal association	(40)

Rows summarize CHM-associated immune mechanisms or proxy measures and intervention-level clinical outcome anchors, including the measured compartment, study system, functional validation, temporal design, and HCC linkage. Clinical outcome anchors inform translational assessment and, where applicable, Tier 3 eligibility; they do not establish immune mediation. (Abbreviations: CD, cluster of differentiation; CCR, C–C chemokine receptor; CHB, chronic hepatitis B; CHM, Chinese herbal medicine; CXCR, C–X–C chemokine receptor; DDC, 3,5-diethoxycarbonyl-1,4-dihydrocollidine; ECM, extracellular matrix; FZHY, Fuzheng Huayu; HBV, hepatitis B virus; HCC, hepatocellular carcinoma; HMGB1, high-mobility group box 1; HSC, hepatic stellate cell; IFN-γ, interferon gamma; IL, interleukin; mDC, myeloid dendritic cell; MMP, matrix metalloproteinase; NAs, nucleos(t)ide analogs; NK, natural killer; NKG2A/NKG2D, natural killer group 2 receptors A/D; NKp46, natural killer cell p46-related protein; NLR, neutrophil-to-lymphocyte ratio; NLRP3, NLR family pyrin domain-containing 3; pDC, plasmacytoid dendritic cell; PSM, propensity score matching; rcccDNA, recombinant covalently closed circular DNA; RCT, randomized controlled trial; Th, T helper; TNF-α, tumor necrosis factor alpha; Treg, regulatory T cell).

**Figure 5:**
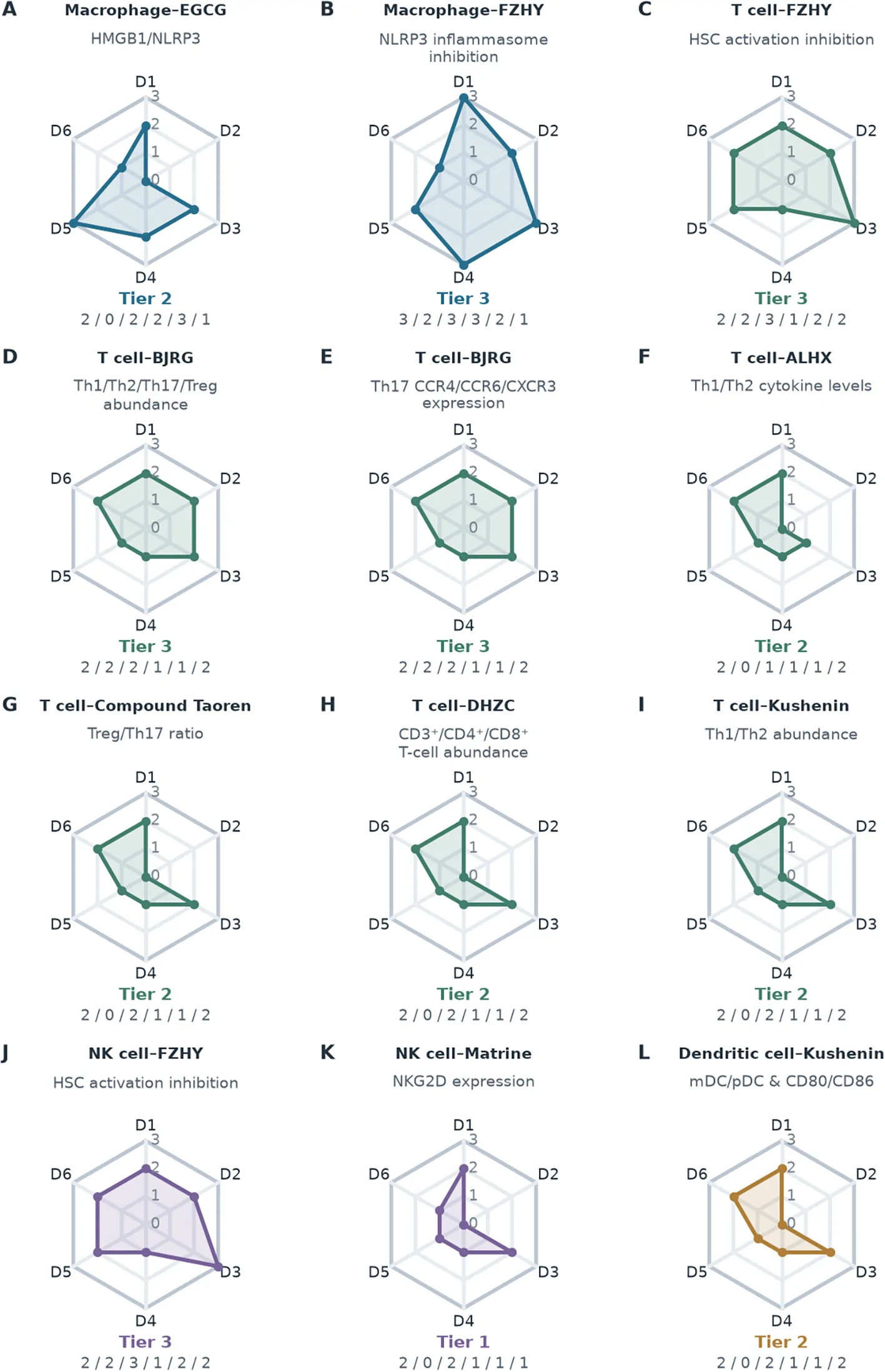
Evidence-maturity profiles and interception tiers of 12 immune-cell–CHM–mechanism combinations. Panels **(A–L)** show combination-specific ratings across six dimensions: D1, pathological relevance; D2, HCC linkage and temporal alignment; D3, evidence level; D4, sampling-compartment directness; D5, functional validation; and D6, translational potential. Scores range from 0 (none) to 3 (high), according to **Table 1**; the numerical sequence beneath each panel follows D1–D6. Colors distinguish immune-cell categories. Each combination is assessed independently, without inheriting disease-side category scores. Tier labels indicate the highest fully satisfied interception threshold defined in **Table 2**. Panel K remains Tier 1 because the documented immune response lacks accompanying fibrosis-related improvement in the assessed evidence set. (CD, cluster of differentiation; CCR, C–C chemokine receptor; CXCR, C–X–C chemokine receptor; HMGB1, high-mobility group box 1; NLRP3, NLR family pyrin domain-containing 3; NKG2D, natural killer group 2 member D; CHM, Chinese herbal medicine; HCC, hepatocellular carcinoma; EGCG, epigallocatechin-3-gallate; FZHY, Fuzheng Huayu; BJRG, Biejia-Ruangan; ALHX, Anluo Huaxian; DHZC, Dahuang Zhechong; HSC, hepatic stellate cell; NK, natural killer; Th, T helper; Treg, regulatory T cell; mDC, myeloid dendritic cell; pDC, plasmacytoid dendritic cell).

### 4.1 Macrophage- and monocyte-associated responses: inflammasome-centered intervention and emerging cancer-interception convergence

Macrophage-associated responses represent one of the more mechanistically resolved components of CHM immunomodulation in HBV-related fibrosis and cirrhosis. Within the CHM intervention literature, the available evidence does not support a uniform shift between conventional “M1” and “M2” phenotypes; instead, it converges more specifically on suppression of macrophage-associated inflammatory signaling and the downstream macrophage–HSC fibrogenic axis. Among these mechanisms, NLR family pyrin domain-containing 3 (NLRP3) inflammasome activity currently emerges as the most consistently supported macrophage-related intervention node, linking hepatocyte-derived danger signals, macrophage inflammatory activation and fibrotic effector responses.

The strongest HBV-specific causal evidence is provided by epigallocatechin-3-gallate (EGCG). In a recombinant covalently closed circular DNA (rcccDNA)-based chronic HBV fibrosis model, EGCG promoted autophagic degradation of cytoplasmic high-mobility group box 1 (HMGB1) in hepatocytes, reduced extracellular HMGB1 and consequently suppressed macrophage NLRP3 inflammasome activation, including caspase-1, IL-1β and IL-18, with parallel attenuation of liver injury and fibrosis (83). Blockade of autophagic flux reversed both inflammasome suppression and the histological benefit, establishing a functional hepatocyte–HMGB1–macrophage NLRP3 sequence and placing the EGCG–HMGB1/NLRP3 axis at Tier 2 as an intervention-responsive immune mechanism. Fuzheng Huayu (FZHY) provides the stronger formulation-level translational bridge. Human HBV fibrotic liver tissue showed reduced NLRP3 expression after treatment, while complementary experiments using a 3,5-diethoxycarbonyl-1,4-dihydrocollidine (DDC)-induced fibrosis model, bone-marrow-derived macrophages, and macrophage-conditioned HSCs linked FZHY to suppression of macrophage NLRP3 activation, inflammatory cytokine release and downstream HSC activation (39). Longitudinal studies of FZHY capsules and tablets have additionally reported lower subsequent HCC incidence (35, 40). Under the intervention–immune mechanism framework, this convergence between macrophage-directed intervention evidence and formulation-level HCC reduction places the FZHY–macrophage NLRP3/HSC axis at Tier 3 as a convergent-evidence cancer-interception candidate.

The current macrophage evidence therefore identifies inflammasome-centered regulation as a principal CHM-responsive mechanism in HBV fibrosis, while also illustrating the remaining gap between pathway-level intervention and cell-state-resolved cancer interception. The macrophage programs associated with later HBV disease progression—including scar-associated CD9/IL18- and CD9/IFI6-defined states—have not yet been mapped directly onto the NLRP3-responsive populations altered by CHM.

### 4.2 T-cell-associated responses: broad immunomodulation with Th17/Treg-centered cancer-interception relevance

T-cell-associated responses constitute the most recurrent immune readouts in clinical CHM studies of HBV-related fibrosis and cirrhosis. The reported effects span several levels of immune resolution, including broad CD3^+^, CD4^+^ and CD8^+^ T-cell composition, helper-T-cell-associated cytokine profiles, Th17/Treg subsets and chemokine-receptor expression. At the lineage level, interventions such as Dahuang Zhechong Capsule plus entecavir increased CD3^+^ and CD4^+^ T-cell measures and the CD4^+^/CD8^+^ ratio while reducing CD8^+^ T-cell measures together with fibrosis-related indices (84). Anluo Huaxian Pill and oxymatrine further showed different helper-T-cell responses: Anluo Huaxian increased IL-2/IFN-γ and reduced IL-4/IL-10, whereas oxymatrine reduced Th1/IFN-γ and increased Th2-related measures (85, 86), indicating that CHM-associated T-cell modulation extends from broad lymphocyte composition to helper-T-cell-related inflammatory regulation. Collectively, these studies establish T-cell immunity as a reproducibly intervention-responsive component of CHM treatment in HBV fibrosis, although the biological information obtained varies substantially with the resolution of the readout.

Among the studies synthesized here, the Th17/Treg-related axis provides the closest correspondence between CHM intervention and the pre-HCC T-cell trajectories defined in Section 3. Biejia-Ruangan plus entecavir was associated with increased circulating Th1 cells and reductions in Th2, Th17 and Treg populations, together with lower inflammatory and collagen-turnover markers (87). A separate 48-week study reported reduced CCR4, CCR6 and CXCR3 expression on circulating Th17 cells in parallel with improvement in fibrosis indices (88), while Compound Taoren Capsule also altered Th17/Treg-related measurements together with inflammatory and fibrotic outcomes (89). Fuzheng Huayu adds complementary functional evidence: patient-derived CD4^+^ T, CD8^+^ T and NK cells collected after treatment altered LX-2 proliferation, apoptosis and profibrotic marker expression in co-culture, demonstrating that treatment-associated lymphocyte remodeling can translate into altered HSC behavior (90). From a cancer-interception perspective, the Biejia-Ruangan–Th17/Treg combination is particularly informative. Section 3 identifies Th17/Treg imbalance as a fibrosis-associated immune state with longitudinal linkage to subsequent HCC, while long-term follow-up of Biejia-Ruangan tablets provides an independent signal of reduced HCC incidence (34). Under the intervention–immune mechanism framework, this convergence places the Biejia-Ruangan–Th17/Treg axis at Tier 3 as a convergent-evidence cancer-interception candidate. The parent placebo-controlled trial additionally demonstrated histological fibrosis regression under the same standardized Biejia-Ruangan intervention, providing the structural-response anchor for this paired evidence chain (32).

The principal gap in the T-cell evidence lies in connecting these peripheral or mixed-lymphocyte responses to the intrahepatic functional states that characterize progression from fibrosis and cirrhosis toward HCC. Most CHM studies measure circulating abundance, cytokines or chemokine receptors, whereas Section 3 indicates that the pre-HCC T-cell landscape is defined more precisely by transitions among effector, regulatory, exhausted and tissue-resident states. The key unresolved correspondence is therefore whether CHM-responsive Th17/Treg changes and broader CD4^+^/CD8^+^ shifts are accompanied by coordinated remodeling of intrahepatic effector and exhausted T-cell populations. The disease-side Th17/Treg–HCC association does not establish that CHM-induced changes in this balance reduce HCC risk.

### 4.3 NK-cell-associated responses: activating-receptor modulation and emerging antifibrotic functional evidence

NK cells occupy a distinctive position in HBV-related fibrosis because their biological functions span both antifibrotic surveillance and tumor immunosurveillance. Among the available interventions, matrine–NKG2D provides the most direct NK-cell-specific pharmacodynamic evidence. In patients with HBV-related cirrhosis, circulating NK-cell NKG2D expression increased after matrine treatment, with concomitant NAs in 43 of 50 patients, whereas NKp46 and the inhibitory receptor NKG2A showed no consistent change (91). The direction of NKG2D modulation corresponds to the disease-associated decline in intrahepatic NKG2D observed with increasing fibrosis severity. However, without accompanying fibrosis-related improvement in the assessed evidence, the pair remains Tier 1. FZHY extends the evidence from receptor phenotype toward immune-cell function. In patients receiving nucleos(t)ide analogs, adjunctive FZHY increased peripheral NK cells and CD4^+^ T cells; treatment-derived CD4^+^ T, CD8^+^ T and NK cells subsequently suppressed LX-2 proliferation, α-smooth muscle actin (α-SMA) and procollagen I expression and promoted HSC apoptosis in co-culture (90). This provides a human functional bridge between CHM-associated lymphocyte remodeling and a fibrotic effector compartment. Independent longitudinal FZHY cohorts have additionally reported lower HCC incidence during antiviral therapy (35, 40). Accordingly, FZHY provides Tier 3 convergent-evidence cancer-interception support at the mixed lymphocyte–HSC level.

The remaining gap is concentrated at the level of intrahepatic NK-cell state and functional specificity. Current CHM studies primarily measure peripheral NK abundance or receptor expression, while the functional FZHY study evaluates several lymphocyte populations. In contrast, the pre-HCC NK landscape described in Section 3 involves redistribution of distinct functional states, including altered activating receptors, cytotoxic programs and NK subsets across fibrosis, cirrhosis and HCC. Paired or longitudinal liver–blood profiling during CHM treatment, combined with NK-cell functional assays, is therefore needed to determine whether the observed peripheral responses reflect restoration of intrahepatic antifibrotic and antitumor NK-cell functions. Independent FZHY studies reporting lower HCC incidence do not establish that these effects are mediated by NK cells.

### 4.4 Neutrophil-associated responses: limited cell-resolved evidence with HCC-linked NLR stratification

Current CHM studies have not directly measured treatment-induced changes in NET formation, neutrophil activation, degranulation or hepatic recruitment. Instead, the principal neutrophil-related clinical signal comes from the NLR, a systemic inflammatory index that integrates both neutrophil and lymphocyte compartments.

A separate FZHY-tablet study reported lower NLR and serum fibrosis markers, without a neutrophil-specific functional assay (92). Among the available studies, FZHY provides the most clinically informative neutrophil-related evidence through NLR-based risk stratification. In a cohort of 916 patients with HBV cirrhosis and no HCC at baseline, baseline NLR stratified both subsequent HCC risk and the association between FZHY treatment and five-year HCC incidence (93). Patients in the medium- and high-NLR strata showed a greater treatment-associated reduction in HCC incidence, indicating that pretreatment inflammatory status may help identify populations with greater apparent benefit from FZHY. This finding supports evaluating NLR for risk stratification and responder enrichment in prospective FZHY studies. Because NLR was used as a baseline stratifier rather than a treatment-responsive neutrophil readout, this evidence represents a clinically informative formulation–inflammation association rather than a cell-resolved neutrophil mechanism.

Thus, the current neutrophil literature already provides a clinically relevant treatment-stratification signal, while the next mechanistic step is to determine whether CHM directly remodels the NET/neutrophil axis itself.

### 4.5 B-cell-, dendritic-cell- and MDSC-associated responses: disease-linked immune axes with limited CHM intervention evidence

In contrast to macrophage-, T-cell- and NK-cell-associated evidence, no clearly resolved CHM intervention–immune mechanism pair has yet emerged for B cells or MDSCs in HBV-related fibrosis or cirrhosis. Available studies have not established CHM-associated changes in defined Breg or other B-cell subsets, or MDSC abundance and suppressive activity together with fibrosis improvement. Accordingly, both axes currently remain at Tier 1: disease-associated candidate immune mechanisms within the interception framework. No B-cell- or MDSC-directed CHM mechanism currently reaches the level of the EGCG–NLRP3 or Biejia-Ruangan–Th17/Treg intervention pairs. By contrast, kushenin-associated myeloid dendritic cell (mDC)/pDC and CD80/CD86 changes accompanied lower serum fibrosis markers, supporting Tier 2 classification without demonstrating antigen-presentation function or immune mediation (86).

These immune-cell populations remain incompletely characterized in CHM intervention studies of HBV-related liver disease. Integrating cell-resolved phenotyping into standardized CHM studies, together with longitudinal assessment of fibrosis and subsequent HCC outcomes, could clarify their intervention responsiveness and cancer-interception relevance.

## 5 Limitations and future directions for immune-mediated incident HCC interception by Chinese herbal medicine

### 5.1 Limitations of the present review

This structured critical review used a mechanism-oriented, representative synthesis. The included studies were selected to resolve immune-cell phenotype or function, anatomical compartment, temporal design and HCC linkage, which facilitates comparison of cancer-interception evidence but gives less weight to studies reporting only broad biochemical or inflammatory outcomes. The main limitation is the separation of the evidence streams. Clinical CHM studies in HBV-related fibrosis mainly characterize peripheral lymphocyte subsets, NK-cell indices or circulating cytokines; higher-resolution macrophage, T-cell and NK-cell studies predominantly address immune or antifibrotic mechanisms; and longitudinal studies of Biejia-Ruangan and Fuzheng Huayu provide incident HCC outcomes without serial cell-resolved immune profiling (31, 34, 35). Thus, the present review can identify convergence between CHM-associated immune modulation and HCC-outcome signals at the intervention level, while the contribution of specific immune changes to HCC-risk reduction remains to be quantified within the same longitudinal population. Variation in CHM formulations, doses, and reported product-characterization details (**Supplementary Table S2**), together with differences in antiviral background, fibrosis definitions, and immune-assay platforms, limits cross-study comparability. Further studies should establish batch reproducibility and dose comparability and assess potential interactions with concomitant nucleos(t)ide analogs.

The Biejia-Ruangan trial extension carries randomized parent-trial allocation, while the FZHY cohorts use matching or NLR-stratified comparisons (**Supplementary Table S3**). Propensity-score matching adjusts measured baseline differences, whereas the NLR analysis evaluates prognostic stratification.

A second limitation arises from the deliberate emphasis on HBV-specific disease evidence. HBV-related fibrosis and cirrhosis models are less extensively represented than established general fibrosis models, while liver immune architecture varies substantially across etiologies. Human single-cell studies have demonstrated distinct monocyte/macrophage, T-cell and B-cell distributions between alcoholic and HBV-related cirrhosis, as well as etiology-associated macrophage, T-cell, NK-cell and intercellular-communication programs across HBV and non-viral cirrhosis; spatial multi-omics of MASLD/MASH further identifies a characteristic lipid-associated macrophage and fibrotic-niche architecture. while Accordingly, this review prioritized HBV-specific human evidence and HBV-relevant systems for identifying disease-side immune trajectories linked to HCC development. Some studies used non-HBV fibrosis models for functional validation or CHM mechanistic exploration. These models provide supportive mechanistic insights but were not considered direct evidence for HBV-specific causal relationships. This strategy preserves etiological relevance but narrows the experimental evidence available for some immune mechanisms. Greater use of HBV-related fibrosis and cirrhosis models will therefore be important for validating CHM-responsive candidate targets identified in general fibrotic settings and for extending their evaluation toward the pre-HCC stage.

For long-term preventive applications, safety assessment should be specific to the preparation, dosage, and exposure duration. Some herbal products have been associated with drug-induced liver injury, highlighting the need for careful quality control and pharmacovigilance (94). Reports of aristolochic-acid-associated mutational signatures in liver cancer further emphasize the importance of avoiding aristolochic-acid-containing ingredients in preventive settings (95). Prospective follow-up should record liver and renal function, adverse events, treatment interruptions, and concomitant medication use, including potential interactions with NAs and other therapies.

### 5.2 Minimum study design required to close the evidence chain for immune-mediated cancer interception

Separate studies have reported CHM-associated antifibrotic effects, immune modulation and lower HCC incidence. The next evidentiary threshold is to connect treatment-induced immune trajectories with incident HCC outcomes within the same pre-HCC population. Using the Fuzheng Huayu–macrophage NLRP3/HSC axis as an example, a multicenter randomized, placebo-controlled, mechanism-embedded study could enroll patients with HBV-related advanced fibrosis or compensated cirrhosis, no baseline HCC, and stable guideline-based antiviral therapy. Participants would receive Fuzheng Huayu plus NA or placebo plus the same NA regimen.

Where randomized trials are not feasible, observational comparisons of adjunctive CHM could emulate a target trial, aligning eligibility, treatment assignment and follow-up to reduce avoidable biases and following the TARGET reporting guidance (96, 97). Parallel experiments should preserve the same CHM, immune target, and pre-tumor intervention window. Treatment would begin after an HBV-related fibrosis model reaches prespecified advanced fibrosis while remaining at the pre-HCC stage. Early sampling would quantify macrophage NLRP3 target engagement, intermediate sampling would assess HSC activity and fibrosis remodeling, and the remaining animals would be followed to tumor development. Macrophage depletion, NLRP3 blockade, and rescue experiments could establish the causal contribution of this axis. Clinical analyses would first estimate the treatment effect on incident HCC and then evaluate the pathways CHM → immune remodeling→ incident HCC and CHM → immune remodeling→ fibrosis remodeling→ incident HCC. Together, randomized treatment effects, serial pre-HCC immune and fibrosis measurements, and experimental perturbation would provide stronger evidence for immune-mediated cancer interception. The minimum paired clinical–experimental design is summarized in **Table 7**.

**Table 7:** Minimum paired clinical–mechanistic design for closing the immune-mediated incident-HCC evidence chain.

Time point	Clinical study	Parallel mechanistic study
T0	Exclude baseline HCC; randomize FZHY + NA vs placebo + the same NA regimen; record baseline covariates, virology, liver stiffness, fibrosis indices and immune panel	Randomize after the HBV-related model reaches prespecified advanced fibrosis while remaining at the pre-HCC stage
Week 12	Repeat blood cell counts/proportions and target-linked NLRP3 assays	Early target-engagement assessment
Week 24	Prespecified primary immune-response assessment using the same panel	Immune-pathway perturbation and/or rescue
Week 48	Repeat immune panel, liver stiffness and serum fibrosis indices; histology where clinically available	HSC activity and fibrosis assessment
Week 96	Main fibrosis assessment using the same measures	—
Every 6 months for 5–7 years	Ultrasound + AFP; confirm suspected HCC by CT/MRI or pathology and blinded adjudication	Follow remaining animals to tumor development
If incident HCC is resected	Exploratory analysis of non-tumor liver tissue for macrophage NLRP3 and HSC activity	–
Throughout follow-up	Record exposure/adherence, concomitant medicines, liver/renal function, adverse events and interruptions	Monitor exposure and tolerability
Prespecified analysis	ITT HCC effect; longitudinal mediation through immune/fibrosis trajectories; baseline/time-updated covariates and sensitivity analyses	Relate target perturbation/rescue to fibrosis and subsequent tumor outcomes

### 5.3 Future directions

Future studies should prioritize immune mechanisms for which disease-side and CHM intervention evidence already converge, particularly macrophage/monocyte–HSC interactions, T-cell differentiation, and NK-cell antifibrotic activity. Multicenter prospective studies can further harmonize antiviral status, fibrosis staging, immune measurements, CHM exposure, and incident HCC surveillance, while recent advances in myeloid metabolism and multicellular fibrotic niches provide additional mechanistic targets for defining how these pathways evolve during fibrosis progression, regression, and transition toward the pre-HCC state (98–100).

Artificial intelligence (AI) may support future integration of longitudinal immune, fibrosis, virological and treatment-exposure data. Existing studies have explored HCC risk prediction in HBV populations using clinical variables and computed tomography (CT)-derived imaging features (101–103). However, machine learning has not consistently outperformed conventional statistical models (104), supporting comparison with established predictors and evaluation of calibration, external validity and clinical utility. Digital traditional Chinese medicine (TCM) phenotypes could be considered as exploratory inputs (105, 106), with their incremental value requiring validation in HBV populations.

Model reporting and appraisal should follow TRIPOD+AI and PROBAST+AI (107, 108). These approaches may help characterize patient trajectories and generate hypotheses, but predictive performance alone cannot establish that CHM-associated immune changes mediate lower HCC risk.

## 6 Conclusion

Among patients with CHB and advanced fibrosis or compensated cirrhosis receiving antiviral therapy, residual heterogeneity in immune–fibrotic remodeling continues to shape incident HCC risk. The evidence synthesized in this review highlights Th17/Treg imbalance, macrophage–HSC inflammatory crosstalk, and impaired NK-cell antifibrotic surveillance as the immune mechanisms showing the greatest convergence across HBV-related fibrosis, CHM responsiveness, and HCC-relevant evidence. Selected standardized CHM formulations further provide clinical evidence of histological fibrosis regression together with signals of reduced HCC incidence. Collectively, these findings support a biologically coherent cancer-interception hypothesis in which CHM may modify actionable immune–fibrotic states during the pre-HCC phase, but CHM-induced immune changes have not been shown to mediate a reduction in HCC incidence.

The next evidentiary threshold is to connect defined CHM exposure, early target-specific immune remodeling, subsequent fibrosis trajectories, and incident HCC outcomes within the same pre-HCC population, supported by intrahepatic anchoring and matched causal experiments. AI-enabled integration of longitudinal clinical, virological, imaging, immune, and digital traditional Chinese medicine phenotypes could further translate these measurements into patient-level immune–fibrotic trajectories for state recognition, treatment-response prediction, incident HCC risk estimation, and individualized assessment of incremental CHM benefit. Building on effective viral suppression, this framework shifts attention toward measurable remodeling of the residual host environment and provides a translational path toward biologically informed CHM intervention, risk-adapted HCC surveillance, and prospective testing of incident HCC interception.

## Glossary

2-AAF: 2-acetylaminofluorene
iNOS: inducible nitric oxide synthase
AI: artificial intelligence
ITGA2B: integrin subunit alpha 2b
ARG1: arginase 1
JUNB: JunB proto-oncogene
BDCA: blood dendritic cell antigen
KLRG1: killer cell lectin-like receptor G1
Breg: regulatory B cell
LAG3: lymphocyte-activation gene 3
C1QA: complement C1q A chain
LX-2: human hepatic stellate cell line
CCl₄: carbon tetrachloride
M1/M2: conventionally polarized macrophage phenotypes
CCL: C-C motif chemokine ligand
MASLD: metabolic dysfunction-associated steatotic liver disease
CCR: C-C motif chemokine receptor
MASH: metabolic dysfunction-associated steatohepatitis
CD: cluster of differentiation
MDSC: myeloid-derived suppressor cell
CD5L: CD5 molecule-like
MMP: matrix metalloproteinase
cDC: conventional dendritic cell
MPO: myeloperoxidase
CHB: chronic hepatitis B
NA: nucleos(t)ide analog
CHM: Chinese herbal medicine
NET: neutrophil extracellular trap
COX: cyclooxygenase
NK: natural killer
CT: computed tomography
NKG2A: natural killer group 2 member A
CTLA-4: cytotoxic T-lymphocyte-associated protein 4
NKG2D: natural killer group 2 member D
CXCL: C-X-C motif chemokine ligand
NKp46: natural cytotoxicity receptor NKp46
CXCR: C-X-C motif chemokine receptor
NLR: neutrophil-to-lymphocyte ratio
DC: dendritic cell
NLRP3: NLR family pyrin domain-containing 3
DDC: 3,5-diethoxycarbonyl-1,4-dihydrocollidine
PAX5: paired box 5
DNA: deoxyribonucleic acid
pDC: plasmacytoid dendritic cell
ECM: extracellular matrix
PD-L1: programmed death-ligand 1
EGCG: epigallocatechin-3-gallate
PGE₂: prostaglandin E2
FASLG: Fas ligand
PLCG2: phospholipase C gamma 2
FasL: Fas ligand
PSM: propensity-score matching
FZD2: frizzled class receptor 2
rcccDNA: recombinant covalently closed circular DNA
FZHY: Fuzheng Huayu
RCT: randomized controlled trial
GNLY: granulysin
RORC: RAR-related orphan receptor C
HBeAg: hepatitis B e antigen
sCD163: soluble cluster of differentiation 163
HBsAg: hepatitis B surface antigen
scRNA-seq: single-cell RNA sequencing
HBV: hepatitis B virus
TCM: traditional Chinese medicine
HCC: hepatocellular carcinoma
TGF: transforming growth factor
HLA-DR: human leukocyte antigen-DR
Th: T helper
HMGB1: high-mobility group box 1
TLR: Toll-like receptor
HSC: hepatic stellate cell
TNF: tumor necrosis factor
IFN: interferon
TRAIL: TNF-related apoptosis-inducing ligand;IFNG, interferon gamma gene
Treg: regulatory T cell;IGLC1, immunoglobulin lambda constant 1
Wnt5a: Wnt family member 5A
IgD: immunoglobulin D
α-SMA: alpha-smooth muscle actin
IL: interleukin.
