# Supplementary material for: Intercepting hepatocellular carcinoma in chronic hepatitis B: a structured critical review of Chinese herbal medicine and the immune–fibrotic evidence
Source: Front Immunol. 2026 Oct 30;17:1985689. doi: 10.3389/fimmu.2026.1985689 (PMC13760456; doi:10.3389/fimmu.2026.1985689)
Supplement: Supplementary file 1 [file Table1.docx]

**Supplementary Table S1. search strategy**

**Review design and coverage. T**his structured critical review used complementary, modular searches across PubMed, Web of Science Core Collection, China National Knowledge Infrastructure (CNKI), and Wanfang Data for English- and Chinese-language records published from 1 January 2010 to 1 August 2026. The search strategy comprised three linked evidence streams: (i) HBV-related immune mechanisms across fibrosis, cirrhosis, and HCC; (ii) CHM-associated immune and fibrotic modulation; and (iii) HCC-outcome evidence.

**Terminology.** CHM included multi-herb formulae and proprietary preparations, single herbs, herb pairs, Chinese medicine injections, standardized extracts, and chemically defined active constituents. Disease-stage, immune-cell, sampling-compartment, temporal-design, functional-validation, and HCC-linkage fields were adjudicated during evidence extraction rather than inferred from search terms alone.

**A. Search architecture**

| **Search stream** | **Core Boolean logic** | **Purpose** |
| --- | --- | --- |
| Stream 1: disease-side immune mechanisms | HBV AND (fibrosis/cirrhosis OR HCC) AND immune-cell terms | Identifies immune mechanisms in HBV-related fibrosis/cirrhosis and their cross-sectional, longitudinal, or experimental connection to HCC. |
| Stream 2a: immune-resolved CHM intervention | HBV AND (fibrosis/cirrhosis OR HCC) AND CHM AND immune-cell terms | Retrieves CHM studies that directly measure one or more prespecified immune-cell populations. |
| Stream 2b: broad CHM fibrosis/cirrhosis intervention | HBV AND fibrosis/cirrhosis AND CHM; no immune-term gate | Captures lower-cell-resolution clinical and experimental studies for full-text checking of immune endpoints. |
| Stream 3: HCC-outcome anchor | HBV AND HCC AND CHM AND incidence/occurrence/risk terms; no mandatory immune or fibrosis term | Captures incident-HCC or experimental-HCC evidence, including HBV–HCC models without an explicitly reported intermediate fibrotic stage. |
| Supplementary ascertainment | Named-product/constituent searches + backward/forward citation checking | Recovers spelling variants, Chinese proprietary medicines, defined constituents, and outcome studies cited by relevant reviews/meta-analyses. |

**B. Concept blocks**

| **Block** | **Terms and synonyms** |
| --- | --- |
| P — HBV aetiology | HBV; hepatitis B; chronic hepatitis B; CHB; HBsAg; HBx; corresponding Chinese-language synonyms. |
| F — fibrotic disease stage | liver/hepatic fibrosis; fibrotic; liver/hepatic cirrhosis; cirrhotic; hepatic stellate cell/HSC; corresponding Chinese-language synonyms. |
| H — HCC | hepatocellular carcinoma; HCC; hepatocarcinogenesis; liver cancer; corresponding Chinese-language synonyms. |
| I — immune-cell populations | T cells (CD4, CD8, Th1, Th2, Th17, Treg); B cells/Breg/plasma cells; NK cells; neutrophils/NETs; macrophages/Kupffer cells/monocytes; dendritic cells; MDSCs; immune microenvironment; immunomodulation. |
| C — CHM exposure | Traditional Chinese medicine; Chinese herbal medicine; Chinese materia medica; herbal formula/decoction/granule/capsule/tablet; proprietary Chinese medicine; herb pair; Chinese medicine injection; standardized extract; single herb; defined/purified active constituent; named products and constituents listed in section G. |
| O — HCC outcome | incidence; occurrence; development; new-onset; cumulative incidence; risk; prevention; interception; chemoprevention; 肝癌发生; 新发肝癌; 累积发生率; 肝癌风险; 防治肝癌. |

**C. PubMed**

Fields: MeSH and Title/Abstract. Date filter: 2010/01/01 to 2026/08/01.

**P (HBV):**

("Hepatitis B"[Mesh] OR "Hepatitis B, Chronic"[Mesh] OR HBV[Title/Abstract] OR "hepatitis B"[Title/Abstract] OR "chronic hepatitis B"[Title/Abstract] OR CHB[Title/Abstract] OR HBsAg[Title/Abstract] OR HBx[Title/Abstract])

**F (fibrosis/cirrhosis):**

("Liver Fibrosis"[Mesh] OR "Liver Cirrhosis"[Mesh] OR fibros*[Title/Abstract] OR cirrho*[Title/Abstract] OR "hepatic stellate cell*"[Title/Abstract] OR HSC[Title/Abstract])

H (HCC):

("Carcinoma, Hepatocellular"[Mesh] OR "hepatocellular carcinoma"[Title/Abstract] OR HCC[Title/Abstract] OR hepatocarcinogenesis [Title/Abstract] OR "liver cancer"[Title/Abstract])

**I (immune cells):**

(immun*[Title/Abstract] OR "immune microenvironment"[Title/Abstract] OR "T cell*"[Title/Abstract] OR CD4[Title/Abstract] OR CD8[Title/Abstract] OR Th1[Title/Abstract] OR Th2[Title/Abstract] OR Th17[Title/Abstract] OR Treg[Title/Abstract] OR "B cell*"[Title/Abstract] OR Breg[Title/Abstract] OR "plasma cell*"[Title/Abstract] OR "NK cell*"[Title/Abstract] OR "natural killer cell*"[Title/Abstract] OR neutrophil*[Title/Abstract] OR NETs[Title/Abstract] OR macrophage*[Title/Abstract] OR "Kupffer cell*"[Title/Abstract] OR monocyte*[Title/Abstract] OR "dendritic cell*"[Title/Abstract] OR MDSC*[Title/Abstract] OR "myeloid-derived suppressor cell*"[Title/Abstract])

**C (generic CHM exposure; supplemented with named products/constituents in section G):**

("Medicine, Chinese Traditional"[Mesh] OR "Drugs, Chinese Herbal"[Mesh] OR "traditional Chinese medicine"[Title/Abstract] OR "Chinese herbal medicine"[Title/Abstract] OR "Chinese materia medica"[Title/Abstract] OR "Chinese patent medicine"[Title/Abstract] OR "herbal formula*"[Title/Abstract] OR decoction*[Title/Abstract] OR granule*[Title/Abstract] OR capsule*[Title/Abstract] OR tablet*[Title/Abstract] OR "herb pair"[Title/Abstract] OR "herbal pair"[Title/Abstract] OR "Chinese medicine injection"[Title/Abstract] OR "herbal injection"[Title/Abstract] OR "standardized extract"[Title/Abstract])

**O (HCC outcome):**

(inciden*[Title/Abstract] OR occurrence[Title/Abstract] OR development[Title/Abstract] OR "new-onset"[Title/Abstract] OR risk[Title/Abstract] OR prevent*[Title/Abstract] OR intercept*[Title/Abstract] OR chemoprevent*[Title/Abstract])

| **PubMed stream** | **Combination** |
| --- | --- |
| Disease-side immune mechanisms | P AND (F OR H) AND I AND 2010/01/01:2026/08/01[Date - Publication] |
| Immune-resolved CHM intervention | P AND (F OR H) AND I AND (C OR named-CHM block) AND 2010/01/01:2026/08/01[Date - Publication] |
| Broad CHM fibrosis/cirrhosis intervention | P AND F AND (C OR named-CHM block) AND 2010/01/01:2026/08/01[Date - Publication] |
| HCC-outcome anchor | P AND H AND (C OR named-CHM block) AND O AND 2010/01/01:2026/08/01[Date - Publication] |

**D. Web of Science Core Collection**

Field: Topic (TS). Publication years: 2010–2026.

| **Set** | **Web of Science Topic query** |
| --- | --- |
| #1 | TS=(HBV OR "hepatitis B" OR "chronic hepatitis B" OR CHB OR HBsAg OR HBx) |
| #2 | TS=("liver fibros*" OR "hepatic fibros*" OR "liver cirrho*" OR "hepatic cirrho*" OR "hepatic stellate cell*" OR HSC) |
| #3 | TS=("hepatocellular carcinoma" OR HCC OR hepatocarcinogenesis OR "liver cancer") |
| #4 | TS= (immun* OR "immune microenvironment" OR "T cell*" OR CD4 OR CD8 OR Th1 OR Th2 OR Th17 OR Treg OR "B cell*" OR Breg OR "plasma cell*" OR "NK cell*" OR "natural killer cell*" OR neutrophil* OR NETs OR macrophage* OR "Kupffer cell*" OR monocyte* OR "dendritic cell*" OR MDSC* OR "myeloid-derived suppressor cell*") |
| #5 | TS= ("traditional Chinese medicine" OR "Chinese herbal medicine*" OR "Chinese materia medica" OR "Chinese patent medicine" OR "herbal formula*" OR decoction* OR granule* OR capsule* OR tablet* OR "herb pair*" OR "Chinese medicine injection*" OR "herbal injection*" OR "standardized extract*") |
| #6 | TS=(incident* OR occurrence OR development OR "new-onset" OR risk OR prevent* OR intercept* OR chemoprevent*) |

| **Web of Science stream** | **Combination** |
| --- | --- |
| Disease-side immune mechanisms | #1 AND (#2 OR #3) AND #4 |
| Immune-resolved CHM intervention | #1 AND (#2 OR #3) AND #4 AND (#5 OR named-CHM block) |
| Broad CHM fibrosis/cirrhosis intervention | #1 AND #2 AND (#5 OR named-CHM block) |
| HCC-outcome anchor | #1 AND #3 AND (#5 OR named-CHM block) AND #6 |

**E. CNKI**

Fields: Subject or title-keyword-abstract; terms within each concept row were joined with OR (+), and concept rows were joined with AND. Journal articles were retained.

| **CNKI stream** | **Search expression** |
| --- | --- |
| Disease-side immune mechanisms | (篇关摘 = 乙型肝炎 OR 乙肝 OR 慢性乙型肝炎 OR HBV) AND (篇关摘 = 肝纤维化 OR 肝硬化 OR 肝星状细胞 OR 肝细胞癌 OR 肝癌) AND (篇关摘 = T细胞 OR B细胞 OR NK细胞 OR 中性粒细胞 OR 巨噬细胞 OR 库普弗细胞 OR 单核细胞 OR 树突状细胞 OR 髓源性抑制细胞) |
| Immune-resolved CHM intervention | (篇关摘 = 乙型肝炎 OR 乙肝 OR 慢性乙型肝炎 OR HBV) AND (篇关摘 = 肝纤维化 OR 肝硬化 OR 肝星状细胞 OR 肝细胞癌 OR 肝癌) AND (篇关摘 = 中药 OR 中医药 OR 中草药 OR 中药复方 OR 中成药 OR 方剂 OR 单味药 OR 中药注射液 OR 标准化提取物 OR 有效成分 OR 活性成分 OR 药对) AND (篇关摘 = T细胞 OR B细胞 OR NK细胞 OR 中性粒细胞 OR 巨噬细胞 OR 库普弗细胞 OR 单核细胞 OR 树突状细胞 OR 髓源性抑制细胞) |
| Broad CHM fibrosis/cirrhosis intervention | (篇关摘 = 乙型肝炎 OR 乙肝 OR 慢性乙型肝炎 OR HBV) AND (篇关摘 = 肝纤维化 OR 肝硬化 OR 肝星状细胞) AND (篇关摘 = 中药 OR 中医药 OR 中草药 OR 中药复方 OR 中成药 OR 方剂 OR 单味药 OR 中药注射液 OR 标准化提取物 OR 有效成分 OR 活性成分 OR 药对) |
| HCC-outcome anchor | (篇关摘 = 乙型肝炎 OR 乙肝 OR 慢性乙型肝炎 OR HBV) AND (篇关摘 = 中药 OR 中医药 OR 中草药 OR 复方鳖甲软肝 OR 扶正化瘀 OR 软肝颗粒 OR 安络化纤) AND (篇关摘 = 肝细胞癌 OR 肝癌 OR 癌变) AND (篇关摘 = 发生 OR 新发 OR 累积发生率 OR 风险 OR 防治) |

**F. Wanfang Data**

| **Wanfang stream** | **Search expression** |
| --- | --- |
| Disease-side immune mechanisms | (乙型肝炎 OR 乙肝 OR 慢性乙型肝炎 OR HBV) AND (肝纤维化 OR 肝硬化 OR 肝星状细胞 OR 肝细胞癌 OR 肝癌) AND (T细胞 OR B细胞 OR NK细胞 OR 中性粒细胞 OR 巨噬细胞 OR 库普弗细胞 OR 单核细胞 OR 树突状细胞 OR 髓源性抑制细胞) |
| CHM intervention | (乙型肝炎 OR 乙肝 OR 慢性乙型肝炎 OR HBV) AND (肝纤维化 OR 肝硬化 OR 肝星状细胞) AND (中药 OR 中医药 OR 中草药 OR 中药复方 OR 中成药 OR 方剂 OR 单味药 OR 中药注射液 OR 有效成分 OR 活性成分 OR 药对) AND (T细胞 OR B细胞 OR NK细胞 OR 中性粒细胞 OR 巨噬细胞 OR 库普弗细胞 OR 单核细胞 OR 树突状细胞 OR 髓源性抑制细胞) |
| HCC-outcome anchor | (乙型肝炎 OR 乙肝 OR 慢性乙型肝炎 OR HBV) AND (中药 OR 中医药 OR 中草药 OR 具体方药名/成分名) AND (肝细胞癌 OR 肝癌 OR 癌变) AND (发生 OR 新发 OR 累积发生率 OR 风险 OR 防治) |

**G. Named CHM exposure supplement**

**Formulae, proprietary preparations, herb pairs, and injections:** Biejia Ruangan/Fufang Biejia Ruangan/Compound Biejia Ruangan, Ruangan granule, Bielong Ruangan, Fuzheng Huayu, Anluo Huaxian, Zishen Rougan, Rougan Huaxian, Yigan Huashi, Tongluo Ruangan, Ruangan Kangxian, Chaishao Liujun, Xiangsha Liujunzi, Yinzhi Ganfu, Fuzheng Qudu, Heidou Sijun, Shugan Jianpi, Jianpi Huayu, Yiqi Jiedu, Erzhu Jiedu, Yanggan Jian, Yiguanjian, Biejiajian, Wuling capsule, Shenqi Yigan, Dahuang Zhechong, Qianggan capsule, Compound Phyllanthus urinaria, Danshen/Salvia miltiorrhiza injection, Astragalus/Huangqi injection, Shenqi Fuzheng injection, Compound Kushen injection, Yinzhihuang injection, and compound glycyrrhizin/diammonium glycyrrhizinate preparations.

**Single herbs, standardized extracts, and chemically defined constituents:** Salvia miltiorrhiza/Danshen, Astragalus membranaceus/Astragali Radix/Huangqi, Rheum palmatum/Rheum officinale/rhubarb, Curcuma longa/Curcumae Rhizoma, Schisandra chinensis, Glycyrrhiza uralensis/licorice, Sophora flavescens, Phyllanthus urinaria, Ligustrum lucidum, Forsythia suspensa, Paeonia lactiflora, Scutellaria baicalensis, Coptis chinensis, Poria cocos, tanshinones, cryptotanshinone, salvianolic acids, astragalosides, curcumin, emodin, rhein, oxymatrine, matrine, glycyrrhizin/glycyrrhetinic acid, berberine, baicalin, baicalein, paeoniflorin, puerarin, luteolin, quercetin, phillygenin, specnuezhenide, bufalin, mulberroside A, piperine, hypericin, calceolarioside B, andrographolide, saikosaponins, schisandrins, polydatin, resveratrol, icariin, and icaritin.

**H. Retrieval and deduplication audit**

| **Database** | **Recorded retrieval yield** | **Deduplicated audit status** |
| --- | --- | --- |
| PubMed | 968 records | 979 records (Eleven additional records) |
| Web of Science Core Collection | 1,727 records | 1,117 records |
| CNKI | 200 records | 127 records |
| Wanfang Data | 377 records | 364 records |
| Pooled candidate library | 3,272 records | 1,885 records |
