# Supplementary material for: Intercepting hepatocellular carcinoma in chronic hepatitis B: a structured critical review of Chinese herbal medicine and the immune–fibrotic evidence
Source: Front Immunol. 2026 Oct 30;17:1985689. doi: 10.3389/fimmu.2026.1985689 (PMC13760456; doi:10.3389/fimmu.2026.1985689)
Supplement: Supplementary file 2 [file Table2.docx]

**Supplementary Table S2. Formulation identity, composition, and reported quality-control information for the Chinese herbal medicine interventions**

| Preparation and source | Formulation type | Composition | Clinical population / experimental model | dose | Reagent/medicine company |
| --- | --- | --- | --- | --- | --- |
| EGCG He 2023 | Single-compound reagent | Epigallocatechin-3-gallate (EGCG). | Mice:rcccDNA-induced chronic-HBV fibrosis mouse model | in vivo ：EGCG (25 mg/kg; Sigma) | Sigma, Shanghai, China |
| FZHY recipe extract Ping 2024 | Chinese herbal formula | Danshen (Salvia miltiorrhiza Bunge; radix; 8g),  Chongcao (Cordyceps sinensis; mycelia; 4g),  Taoren (Prunus persica Batsch; fruit; 2g),  Jiaogulan (Gynostemma pentaphyllum Makino; whole herb; 6g),  Songhuafen; pinus massoniana Lamb; pollen; 2g,  Wuweizi (Schisandra chinensis Baill; fruit; 2g) | Mice：DDC-induced fibrotic mouse model; Human：HBV-related fibrosis | Human: FZHY + ETV  in vivo: Mice,2.8 or 5.6 g/kg/day | Shanghai Sundise Medicine Technology Development Co. Ltd., China |
| FZHY capsules Wu 2020 | Chinese herbal preparation | Dan Shen (Radix Salviae Miltiorrhizae),  Chong Cao (Paecilomyces hepiali Chen & Dai),  Tao Ren (Semen Persicae),  Jiao Gu Lan (Gynostemma pentaphyllum),  Song Hua Feng (Pinus armandii Franch.),  Wu Wei Zi (Fructus Schisandra chinensis). | Mice:ConA-induced fibrotic mouse model; Human:HBV-related fibrosis | Human: FZHY, 5 capsules (0.3g per capsule), three times daily+antiviral NAs(Entecavir, Lamivudine,or Adefovir for oral administration,  0.5mg, 100mg, or 10mg daily).  in vivo: Mice,20mL/kg | Shanghai Modern Pharmaceutical Company, China |
| FZHY capsules Shi 2020; Shi 2023 | Chinese herbal preparation | Raw materials：  Danshen (Radix salvia miltiorrhizae,666g),  Chongcao (Cordyceps,334g),  Taoren (Semen persicae,166g),  Jiaogulan (Gynostemma pentaphyllum,500g),  Songhuafen (Pollen pini,166g),  Wuweizi (Fructus schisandrae chinensis,166g) | Human:HBV-related cirrhosis | FZHY, 5 capsules (0.3g per capsule)，three times daily+ETV or TDF | Huanghai Pharmaceutical Co., Ltd., Shanghai,  China (the approval number:Z20020073) |
| FZHY tablets Fan 2024 | Chinese herbal preparation | Not provided | Human:HBV-related cirrhosis | FZHY, 1.6 g per dose, three times daily + ETV | Not provided |
| FZHY tablets Cai 2025 | Chinese herbal preparation | Danshen, Chongcao, Taoren, Jiaogulan, Songhuafen, Wuweizi | Human:HBV-related cirrhosis | FZHY, 1.6 g per dose (four 0.4g tablets), three times daily + ETV | Huanghai Pharmaceutical Co., Ltd., Shanghai,  China (the approval number:Z20050546) |
| BJRG tablets Yang 2018 | Chinese herbal preparation | Biejia (turtle shell), Ezhu, Chishao, Danggui, Sanqi, Dangshen, Huangqi, Ziheche, Dongchongxiacao, Banlangen, and Lianqiao | Human:HBV | BJRG, 2 g per dose, three times daily + ETV | Not provided |
| BJRG tablets Gao 2018 | Chinese herbal preparation | Biejia (processed), Ezhu, Chishao, Danggui, Sanqi, Dangshen, Huangqi, Ziheche, Dongchongxiacao, Banlangen, and Lianqiao | Human:HBV-related fibrosis | BJRG, 2 g per dose(0.5 g/tablet), three times daily + ETV | Inner Mongolia Furui Medical Science Co.(the approval number: Z19991011), China |
| BJRG tablets Rong 2022; Ji 2022 | Chinese herbal preparation | Biejia (processed), Ezhu, Chishao, Danggui, Sanqi, Dangshen, Huangqi, Dongchongxiacao, Banlangen, and Lianqiao | Human:HBV-related fibrosis | BJRG, 2 g per dose, three times daily + ETV | The randomized treatment lasted 72 weeks; the cited follow-up derives from the parent trial.  Not provided |
| ALHX pills Jia 2017 | Chinese herbal preparation | Not provided | Human: CHB and HBV-cirrhosis | ALHX, 6 g per dose, three times daily + ETV | Senlong Pharmaceutical (the approval number: Z20010098), China |
| Compound Taoren capsules Li 2023 | Chinese herbal preparation | Dongchongxiacao 5g, Tu Bie Chong 5g, Chaihu 6g; Taoren 10g; Danggui 10g; Huangqi 15g; Chishao 15g; Danshen 30g | Human:HBV-related fibrosis | Compound Taoren, 0.45g/capsule; 3 capsules per dose, three times daily + ETV | Suyaozhizi (the approval number: Z04001331), China |
| DHZC capsules Yao 2019 | Chinese herbal preparation | Not provided | Human:HBV-related cirrhosis | DHZC, 0.4 g/capsule; 4 capsules per dose, twice daily + ETV | Jiangsu Yihai (the approval number Z20054026; batch 170905), China |
| Matrine injection Mao 2016 | Single-constituent clinical injectable preparation | Kushen | Human:HBV-related cirrhosis | Matrine,150 mg/day by intravenous infusion+ oral NAs | Guangzhou Baiyunshan mingxing (the Batch 121101), China |
| Kushenin capsules Zhu 2018 | Single-herb-derived alkaloid preparation | Kushen | Human:HBV | Kushenin, 1 capsule daily + ETV | Not provided |

Information is presented as reported in each cited study. “Not reported” indicates that the information was not specified in the report reviewed.（ALHX, Anluo Huaxian; BJRG, Biejia Ruangan; CHB, chronic hepatitis B; DHZC, Dahuang Zhechong; EGCG, epigallocatechin-3-gallate; ETV, entecavir; FZHY, Fuzheng Huayu; HBV, hepatitis B virus; NAs, nucleos(t)ide analogues）。
