# Supplementary material for: Intercepting hepatocellular carcinoma in chronic hepatitis B: a structured critical review of Chinese herbal medicine and the immune–fibrotic evidence
Source: Front Immunol. 2026 Oct 30;17:1985689. doi: 10.3389/fimmu.2026.1985689 (PMC13760456; doi:10.3389/fimmu.2026.1985689)
Supplement: Supplementary file 3 [file Table3.docx]

Supplementary Table S3. Clinical incident-HCC outcome studies of Chinese herbal formulations

| **Formulation** | **Design / population / comparison** | **Follow-up / HCC assessment** | **Reported HCC outcome** | **Confounding control** |
| --- | --- | --- | --- | --- |
| Biejia-Ruangan tablets Ji 2022 | 14-centre RCT extension; 1,000 treatment-naïve patients with HBV-related fibrosis (Ishak ≥3); 500/group. ETV + BJRG vs ETV + placebo for 72 weeks, then open-label treatment. | 7 years. Ultrasound/AFP every 6 months; contrast-enhanced CT/MRI and biopsy confirmation. | 7-year cumulative incidence(Kaplan–Meier estimates): 4.7% vs 9.3%; HR 0.489 (95% CI 0.288–0.832). Adjusted mITT HR 0.405 (0.230–0.710). | Parent-trial randomization (1:1); multivariable Cox regression including treatment allocation. |
| Fuzheng Huayu capsules Shi 2020 | Single-centre retrospective cohort (2011–2015); 842 patients with HBV-related cirrhosis (270 vs 572); 259 PSM pairs. FZHY + ETV/TDF vs ETV/TDF. | 5 years. AFP/radiology every 3–6 months; histology and/or characteristic multiphasic CT/MRI. | After PSM, 5-year KM cumulative incidence: 10.0% vs 23.2%; aHR 0.32 (95% CI 0.19–0.53). | 1:1 PSM using age/sex, alcohol, diabetes/hypertension, HBV markers, laboratory indices and cirrhosis severity (CTP/MELD); additional multivariable Cox adjustment. |
| Fuzheng Huayu tablets Fan 2024 | 20-centre retrospective cohort (2013–2021); 910 patients with compensated HBV-related cirrhosis (458 vs 452); 231 PSM pairs. FZHY + ETV vs ETV. | Median 39 months; 5-year estimates. Pathology or CT/MRI; surveillance interval not reported. | After PSM, 5-year KM cumulative incidence: 9.8% vs 21.8%. FZHY >36-month subgroup: aHR 0.305 (95% CI 0.113–0.823) | 1:1 PSM using age/sex, HBV markers, liver/renal indices, platelets and AFP; Cox adjustment for age, white blood cell count, platelets, ALT and GGT. |
| Fuzheng Huayu capsules Shi 2023 | Single-centre retrospective cohort (2011–2016); 916 patients with HBV-related cirrhosis (Child–Pugh A–C). FZHY + ETV/TDF (299) vs ETV/TDF (617) | 5 years. Histology or characteristic contrast-enhancement/washout; surveillance interval not reported. | 5-year HCC: 32/299 (10.7%) vs 122/617 (19.8%); comparisons stratified by baseline NLR. | Baseline-NLR spline analysis adjusted for age/sex, HCC family history, diabetes, liver/renal indices, AFP and HBV DNA. |

Notes. This table summarizes the four clinical incident-HCC outcome reports included in Table 5. Intervention groups are listed before comparator groups. Formulation composition, dosing and manufacturer information are provided in Supplementary Table S2. (Abbreviations. AFP, alpha-fetoprotein; aHR, adjusted hazard ratio; ALT, alanine aminotransferase; BJRG, Biejia-Ruangan; CI, confidence interval; CT/MRI, computed tomography/magnetic resonance imaging; CTP, Child–Turcotte–Pugh; ETV, entecavir; FZHY, Fuzheng Huayu; GGT, gamma-glutamyl transferase; HBV, hepatitis B virus; HCC, hepatocellular carcinoma; HR, hazard ratio; KM, Kaplan–Meier; MELD, model for end-stage liver disease; mITT, modified intention-to-treat; NLR, neutrophil-to-lymphocyte ratio; PSM, propensity-score matching; RCT, randomized controlled trial; TDF, tenofovir disoproxil fumarate).
